# Synthesizing a Hybrid Nanocomposite as an Affinity Adsorbent through Surface-Initiated Atom Transfer Radical Polymerization Catalyzed by Myoglobin

**DOI:** 10.1021/acsomega.1c00955

**Published:** 2021-04-12

**Authors:** Solmaz Hajizadeh, Leif Bülow, Lei Ye

**Affiliations:** Division of Pure and Applied Biochemistry, Department of Chemistry, Lund University, 22100 Lund, Sweden

## Abstract

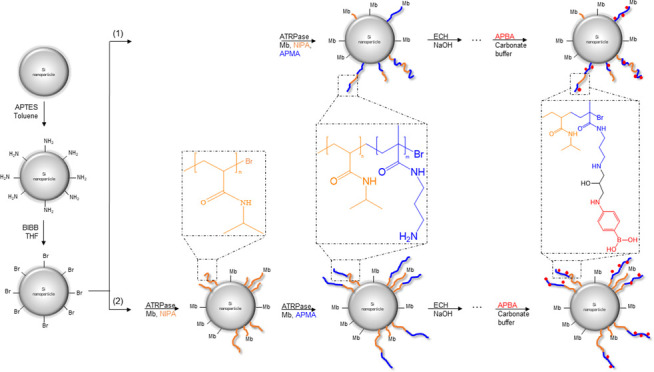

A hybrid bifunctional core–shell nanostructure was synthesized for the first time via surface-initiated atom transfer radical polymerization (SI-ATRP) using myoglobin as a biocatalyst (ATRPase) in an aqueous solution. *N*-Isopropyl acrylamide (NIPA) and *N*-(3-aminopropyl)methacrylamide (APMA) were applied to graft flexible polymer brushes onto initiator-functionalized silica nanoparticles. Two different approaches were implemented to form the core–shell nanocomposite: (a) random copolymerization, Si@p(NIPA-*co*-APMA) and (b) sequential block copolymerization, Si@pNIPA-*b*-pAPMA. These nanocomposites can be used as versatile intermediates, thereby leading to different types of materials for targeted applications. In this work, a phenylboronic acid ligand was immobilized on the side chain of the grafted brushes during a series of postmodification reactions to create a boronate affinity adsorbent. The ability to selectively bind glycoproteins (ovalbumin and glycated hemoglobin) via boronic acid was assessed at two different temperatures (20 and 40 °C), where Si@pNIPA-*b*-APMA_BA_ (163 mg OVA/g of particle) displayed an approximately 1.5-fold higher capacity than Si@p(NIPA-*co*-APMA)_BA_ (107 mg OVA/g of particle). In addition to selective binding to glycoproteins, the nanocomposites exhibited selective binding for myoglobin due to the molecular imprinting effect during the postmodification process, that is, 72 and 111 mg Mb/g for Si@p(NIPA-*co*-APMA)_BA_ and Si@pNIPA-*b*-pAPMA_BA_, respectively.

## Introduction

Since the introduction of atom transfer radical polymerization (ATRP), this method has been utilized to form complex topologies using metal ions as catalysts.^1^ The technique enables the manipulation of molecular weight with low polydispersity.^2^ Being able to control the polymerization reaction, the commercial availability of reaction components, such as catalysts, monomers, and initiators, and the possibility of stopping and restarting the reaction are some of the advantages of the ATRP system.^1,2^ In addition, ATRP can be carried out in aqueous systems, which makes it green in comparison to traditional systems.^3,4^ The surface-initiated atom transfer radical polymerization (SI-ATRP) approach is one of the subcategories of ATRP.^5^ This method enables the synthesis of well-defined polymer brushes on biological and inorganic surfaces^6−9^ for various applications, such as drug delivery, tissue engineering, biosensors, and bioseparation.^10^ This procedure can be performed under mild conditions, and by using chemicals that respond to external stimuli, such as temperature and pH, “smart” materials can be formed. Considering all of the advantages of the ATRP reaction, consuming a high concentration of catalyst could be cited as a disadvantage. Therefore, many recent studies have focused on reducing catalyst consumption to the ppm level, where external physical or chemical forces regenerate the active parts.^11,12^ This approach makes the system more environmentally friendly and economical. However, even with this improvement, the risk of having an inorganic catalyst present in the formed product makes this approach less interesting for many applications, such as biomedical purposes. To address this challenge, Bruns’ group has used hemoproteins, such as horseradish peroxidase^13^ and hemoglobin (Hb),^14^ for the first time as ATRP catalysts (ATRPases) to form free chain polymers. The concept of growing polymer brushes on a surface using metalloenzymatic radical polymerization was reported in the literature,^13,15−18^ where catalase from bovine liver and laccase from *Trametes versicolor* were utilized as biocatalysts to graft poly(*N*-isopropyl acrylamide) (pNIPA) polymer brushes from nanofiber surfaces.^15^

**Figure 1:**
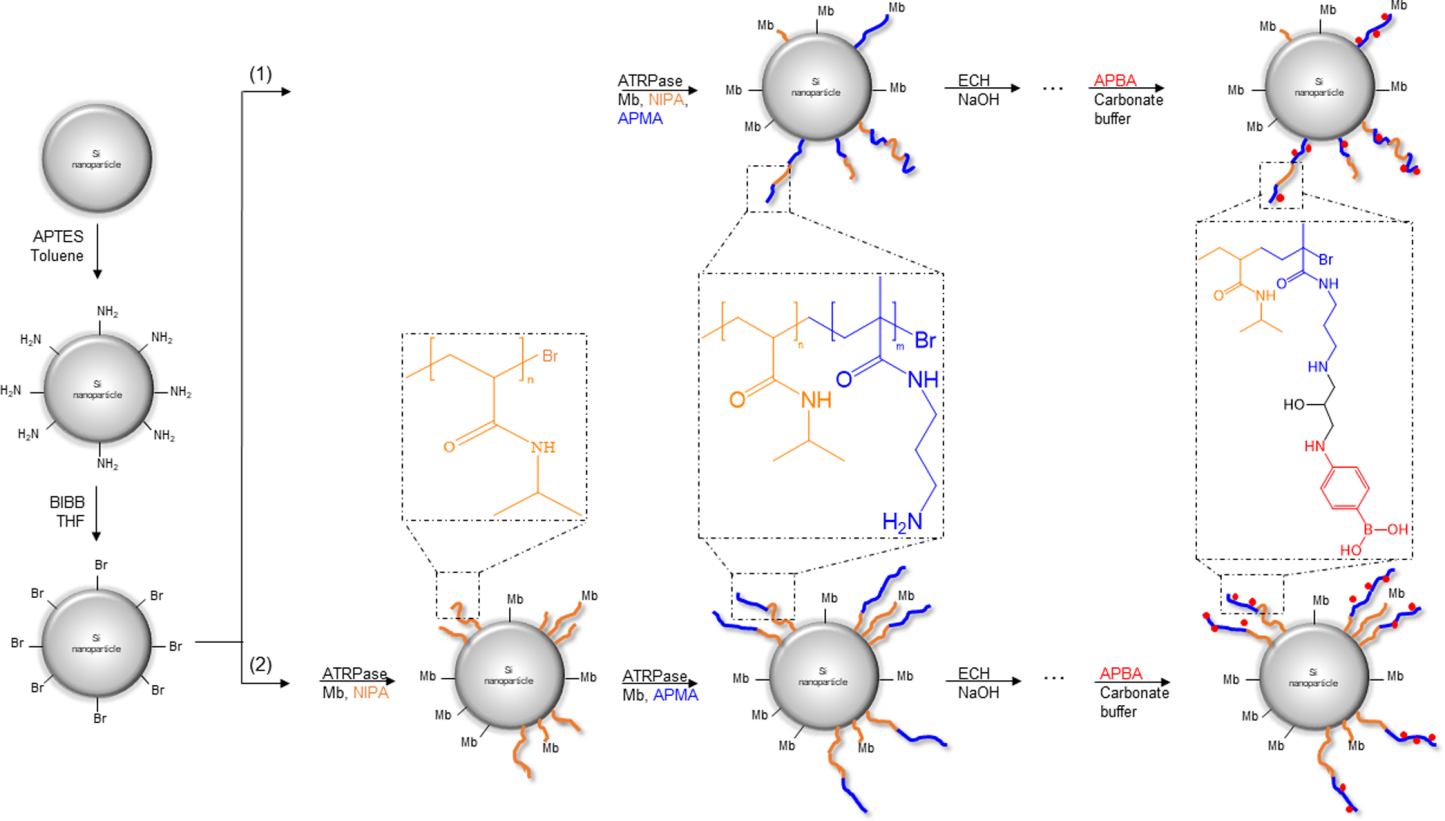
Schematic formation of nanocomposite via an ATRP reaction using Mb as a catalyst.

In this study, a new approach to form a hybrid core–shell nanocomposite on silica nanoparticles using myoglobin, Mb, as a catalyst (SI-ATRPase) was evaluated. Poly(*N*-isopropyl acrylamide) (pNIPA) and poly(*N*-(3-aminopropyl)methacrylamide) (pAPMA) were grafted in two different arrangements from functionalized silica nanoparticles to produce smart nanocomposites. The two different approaches are (1) a one-step SI-ATRP to form random copolymer brushes and (2) a two-step SI-ATRP to create sequential block copolymer brushes (Figure 1).

The engineered material as a boronate affinity adsorbent was assessed in this work as an application model for the particles. For this purpose, phenylboronic acid (PBA) was attached to the side chain of pAPMA via an available amine group during a series of postmodifications. Two different glycated proteins, ovalbumin (OVA) and glycated hemoglobin (HbA1c), and bovine serum albumin (BSA) as a negative control, were selected to study the adsorption/elution capability of the appended boronic ligand on the grafted brush. The impact of the temperature on the adsorption process was investigated.

The postmodification of the nanocomposite was conducted by epichlorohydrin (a cross-linker) in the presence of Mb. As a result, pseudomolecularly imprinted polymer (p-MIP) particles were formed unintentionally during postmodification. The selectivity of the MIP particles was assessed toward Mb in the presence of BSA.

## Results and Discussion

### Formation of Si@p(NIPA-*co*-APMA)_BA_ and Si@pNIPA-*b*-pAPMA_BA_ Core–Shell Nanocomposites

The conventional Stöber process was applied to form silica nanoparticles, and amino groups were placed on the surface of the silica nanoparticles using (3-aminopropyl)triethoxysilane (APTES). Dynamic light scattering (DLS) was used to measure the size of the particles in different stages. The average diameters of the silica nanoparticles before and after introducing amine groups on the surface were 180 ± 5 nm (polydispersity index (Pdl): 0.036) and 190 ± 10 nm (Pdl: 0.212), respectively. Si@NH_2_ nanoparticles were subjected to 2-bromoisobutyryl bromide (BIBB) to form Si@initiator. As a result, the size of the nanoparticles was 210 ± 3 nm (Pdl: 0.301). The effect of the introduction of amine groups followed by the initiator on the silica nanoparticles can be seen in the Fourier transform infrared (FTIR) spectra (Figure S1). The asymmetric stretching vibrations of the Si–O–Si band can be detected at 1053 cm^–1^ in Figure S1. The changes at 3300 cm^–1^ are explained by the introduction of amino groups on the nanoparticles by the APTES reaction. The reduction in the band intensity at 3300 cm^–1^ occurred after introducing the Br initiator on the amine groups. Two absorption peaks appeared at 1640 and 1490 cm^–1^, which were associated with amide I and amide II after adding the ATRP initiator (Figure S1).

The morphology and particle size of the Si nanoparticles, Si@NH_2_ and Si@initiator, were evaluated from scanning electron microscopy (SEM) images (Figure S2). Based on the images, the particles have a homogeneous size distribution, and there is no significant difference between the core nanoparticles before and after introducing the initiator. The silica core has a smooth surface, and its diameter was measured at 214 and 249 nm (using ImageJ 1.52a) before and after modification, respectively.

In this paper, and for the first time, the formation of silica core–shell nanocomposites using ATRPase was examined. Two different types of polymer brushes were grafted successfully from Si@initiator nanoparticles using Mb as a catalyst: (1) random copolymerization for Si@p(NIPA-*co*-APMA) and (2) block copolymerization for Si@pNIPA-*b*-pAPMA. NIPA and APMA were selected as monomers for this study due to their water solubility. Thus, it was possible to conduct the ATRP reaction in aqueous conditions without applying any organic solvent. NIPA is a well-known thermoresponsive monomer with a lower critical solution temperature (LCST) of 32 °C. APMA also belongs to the acrylamide family with a free amine group as a side chain. In the first approach, random copolymer brushes of pNIPA and pAPMA were formed on the functionalized silica-based core in a one-step polymerization. In the second approach, grafting was performed in two steps. First, a layer of pNIPA was grafted from the core nanoparticles, and then a second block, pAPMA, was grafted from the pNIPA brushes. Figure 1 illustrates the schematic of the two different approaches.

**Figure 2:**
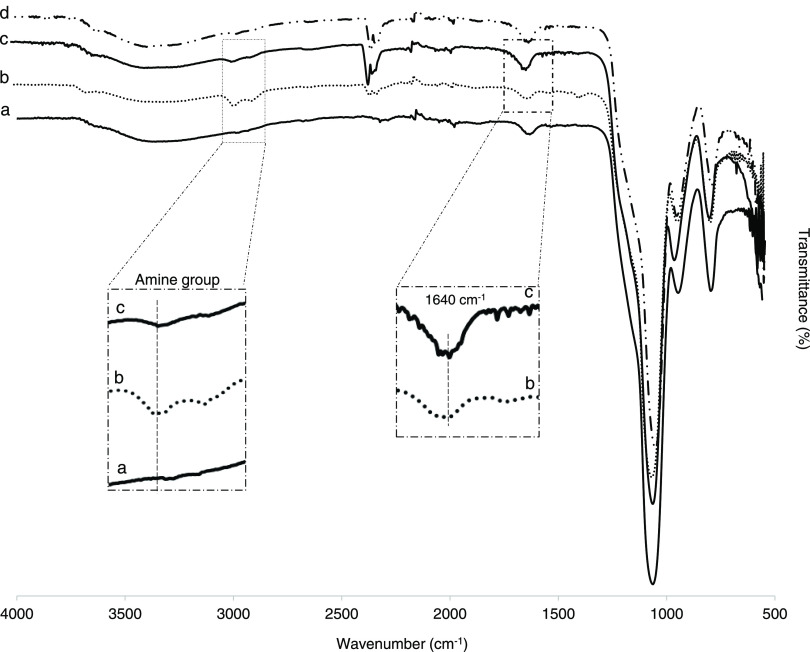
FTIR spectra of (a) Si@initiator, (b) Si@p(NIPA-*co*-APMA), (c) Si@ p(NIPA-*co*-APMA)_ep_, and (d) Si@p(NIPA-*co*-APMA)_BA_.

**Figure 3:**
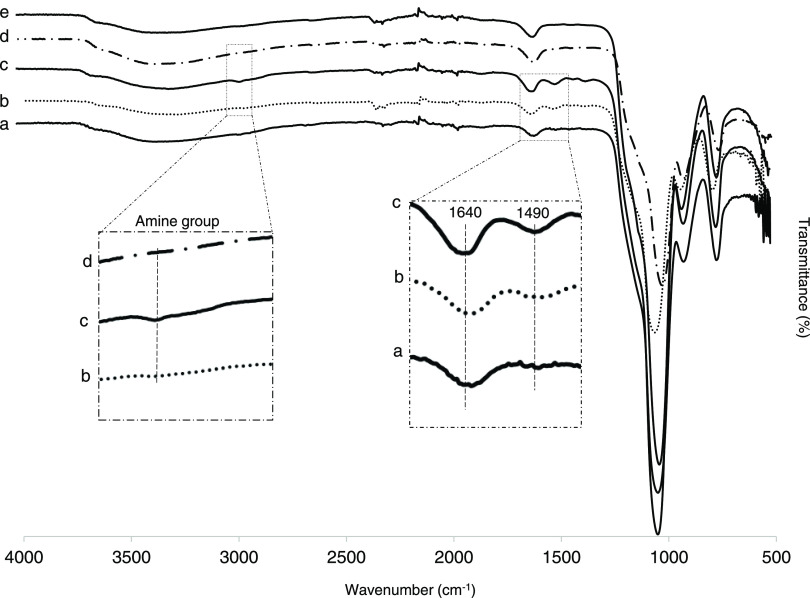
FTIR spectra of (a) Si@initiator, (b) Si@pNIPA, (c) Si@pNIPA-*b*-pAPMA, (d) Si@pNIPA-*b*-pAPMA_ep_, and (e) Si@pNIPA-*b*-pAPMA_BA_.

FTIR spectra of the Si@p(NIPA-*co*-APMA) and Si@pNIPA-*b*-pAPMA particles are presented in Figures 2 and 3, respectively. From Figure 2 (spectra a and b), the intensities of the amide I and amide II bands at 1640 and 1490 cm^–1^, respectively, increased by introducing the grafted brush. In addition, new bands at approximately 3000 cm^–1^ appeared, which is indicative of free amino groups on the APMA polymer chain. The same pattern was observed for the block copolymer particle, where the intensity of the amide I (1640 cm^–1^, C=O stretching) and amide II (1490 cm^–1^, N–H stretching) signals increased after introducing the second layer of the polymer brush, which is a sign of successful sequential grafting of the polymer brush under two-step ATRP reactions (Figure 3).

A control experiment was designed to study the effect of iron in the heme group on the ATRPase reaction. Both Fe(II) and Fe(III) have been utilized as catalysts in ATRP reactions.^19^ In the case of using heme protein by Bruns’ group, it was suggested that carbon-centered radicals are created when Fe(II) abstracts Br from the initiator, which then starts chain propagation.^14^ In a control experiment, first, the iron atom was blocked by cyanide due to its high affinity. Cyanide can interact with both ferric and ferrous heme proteins.^20^ This treatment stopped the formation of Fe(III)–Br radicals and, as a consequence, the formation of the pNIPA chain. Figure S3A shows the spectra of Mb before and after reaction with cyanide, where the characteristic peak of the protein shifted from 409 to 420 nm^21^ when the protein changed from the ferric state (Fe^3+^) to the cyano met-Mb form. The reaction outcomes for both setups, control and regular, are presented in Figure S3B,C. The thermoresponsiveness of the free polymer chain can be observed when commercial Mb is used directly as a catalyst. Blocking the heme group with cyanide prevents the ATRPase reaction, and therefore, no visual or physical changes can be followed by changing the temperature.

Epichlorohydrin was used to introduce epoxy rings on the free amino groups of the polymer brush. The modification was followed by introducing an aminophenyl boronic acid ligand on the pendant epoxy group under alkaline conditions. Disappearance of the amine group at approximately 3000 cm^–1^ can be a sign of a successful reaction (Figure 2, spectra b and c). The same trend of appearing and disappearing amine groups after grafting pAPMA and introducing epoxy rings is shown in Figure 3 (spectra b and c). Antisymmetric stretching of the epoxy ring has a weak signal, which appears at 856 and 909 cm^–1^.^22^ From the FTIR spectra of both nanocomposites, it was not possible to detect the epoxy peaks. The absorption peak of the B–O band after reaction with aminophenyl boronic acid was also not clear from the spectra. Therefore, elemental analysis was performed to confirm that the reaction was successful after each step of the modification (Table 1).

**Table 1:** Elemental Analysis Results

	sample	%C	%H	%N	%Br	%B	Fe (ppm)
1	Si@NH_2_	3.00 ± 0.01	2.06 ± 0.01	0.77 ± 0.05	n.a.	n.a.	n.a.
2	Si@initiator	3.70 ± 0.02	2.19 ± 0.01	0.79 ± 0.01	3.40 ± 0.01	n.a.	n.a.
3	Si@pNIPA	4.47 ± 0.01	2.12 ± 0.01	0.88 ± 0.01	0.48 ± 0.01	n.a.	419 ± 2
4	Si@p(NIPA-*co*-APMA)	4.65 ± 0.01	2.93 ± 0.01	0.84 ± 0.01	0.44 ± 0.01	n.a.	465 ± 2
5	Si@pNIPA-*b*-pAPMA	7.84 ± 0.01	2.38 ± 0.01	1.88 ± 0.01	0.44 ± 0.01	n.a.	809 ± 2
6	Si@p(NIPA-*co*-APMA)_BA_	4.12 ± 0.01	2.34 ± 0.01	0.70 ± 0.01	<0.01	0.05 ± 0.004	30 ± 5
7	Si@pNIPA-*b*-pAPMA_BA_	6.10 ± 0.01	2.77 ± 0.01	1.60 ± 0.01	<0.01	0.10 ± 0.030	50 ± 5

Based on the elemental analysis, the weight percentage of the CHN increased after introducing a polymer brush on silica nanoparticles (Table 1, samples 3–7). However, the rise in the percentage of CHN is not very high compared to the traditional ATRP reaction using inorganic catalysts. For example, Si@pNIPA particles formed via traditional ATRP have approximately 9, 3, and 1% C, H, and N, respectively.^23,24^ By adding the second layer to the polymer brush, the %C can reach up to 34%.^24^ The different reaction conditions may explain the low density of the grafted polymer brush in this work. It was reported that bromoacetate (and iodoacetate) reacts with several amino acids, such as cysteine (via sulfhydryl group), histidine (via imidazole side-chain), methionine (via thioether), lysine (via ε-amino group), and the N-terminal amino group.^25^ Mb has 153 amino acid residues and an iron located at the center of a heme group, where it interacts with four nitrogen atoms, an imidazole side chain of His-64, and an oxygen molecule.^26,27^ The other likelihood is the nonspecific interaction of the protein with Si@Br which prevents the initiator from participating in the reaction. Thus, while the iron atom in Mb acts as a catalyst in the ATRPase reaction, the protein can covalently or noncovalently attach to the surface of the particles and hinder the reaction process. These interactions make it difficult for the polymer brush to grow homogeneously from the core. Therefore, the %CHN of the particles after polymerization is not as high as expected (Table 1). However, after grafting random copolymer and block polymer onto the silica nanoparticles, some bromide is available and intact, which allows the process to continue (Table 1).

**Figure 4:**
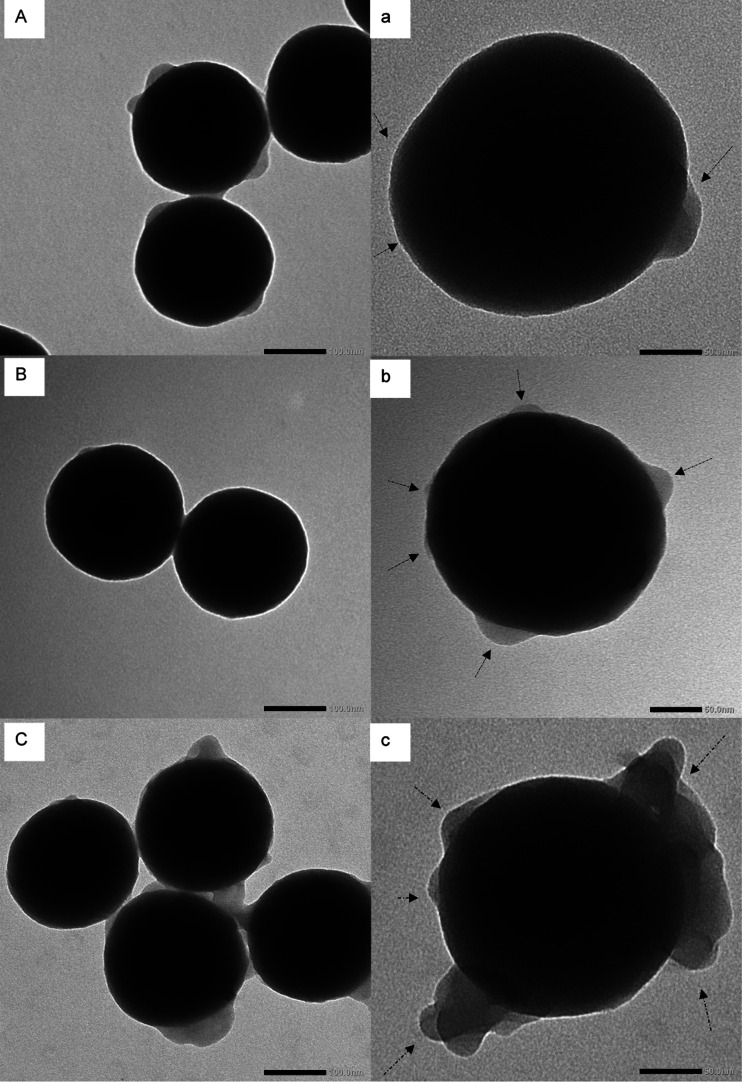
TEM images of (A) Si@pNIPA nanocomposite, (B) Si@p(NIPA-*co*-APMA) nanocomposite, and (C) Si@pNIPA-*b*-pAPMA nanocomposite (scale bar is 100 nm). Images (a)–(c) are the higher magnifications of (A)–(C), respectively, with a scale bar of 50 nm. The arrows pointed toward the visible polymer brushes formed on the surface of silica nanoparticles.

Transmission electron microscopy (TEM) images (Figure 4) illustrate the irregular shape of the grafted brush on the silica core. The uneven formation of the polymer brushes on the surface of the silica core may be explained by physical or chemical blockage of the initiator during the ATRPase reaction. The pNIPA-grafted brush on silica nanoparticles as well as a random polymerization of NIPA and APMA formed small bumps (diameter ∼10 nm, measured by ImageJ) on the surface of the nanoparticles. The block copolymer-grafted brushes on the silica nanoparticles are more visible in TEM images (Figure 4c). These shells also appeared asymmetric around the core nanoparticles (Figure 4C,c), but in comparison to Figure 4a, the brushes were larger (20–30 nm in diameter). Therefore, the content of %CHN is slightly higher for the Si@pNIPA-*b*-pAPMA nanocomposite, which can be expected based on the TEM images. It should be mentioned that there is a possibility that polymer brushes of two different particles attach during the ATRPase reaction while growing (Figure 4A–C). Reaction conditions, such as the agitation method and its speed, can play a role in this phenomenon. The aggregation of the particles can limit the growth of the polymer brushes and disturb the postmodification.

Based on the TEM images, it is difficult to conclude what percentage of the surface has been covered by the polymer brushes (Figure 4a,b). Therefore, elemental mapping of the nanocomposites was conducted using SEM-energy-dispersive spectrometry (EDS) (Figures S4 and S5). Based on carbon mapping of random and block copolymer particles, the distribution of the element can be followed on the surface of the silica core (purple dots inside the marked area in Figures S4C and S5C). The presence of carbon can be an indication that the grafted polymer brushes cover the surface of the silica nanoparticles in both approaches. An analysis of nitrogen mapping showed that the density of this element is slightly higher on the particle surface than in the surrounding area (pink dots inside the selected area in Figures S4D and S5D). However, due to the low concentration, the element mapping is mainly disturbed by the noise level. This finding is in keeping with the elemental analysis (Table 1), which indicates a low concentration of nitrogen in the shell. The elemental mappings for silica and oxygen are powerful due to their high concentrations (Figures S4 and S5E,F). On the other hand, due to the low concentration of iron and bromide on the surface of the particles (Table 1), there were no notable differences in the mapping analysis, and the distribution of these elements was disturbed by the background noise (Figures S4 and S5G,H). The energy-dispersive X-ray (EDX) spectra of the Si@p(NIPA-*co*-APMA) and Si@pNIPA-*b*-pAPMA nanocomposites are shown in Figure S6. The spectra reveal that the peaks related to bromide, iron, and nitrogen are very weak and hidden in the background noise.

Based on the elemental analysis, the content of iron in the Si@pNIPA-*b*-pAPMA nanocomposite is 0.246 mg Mb/mg particle after synthesis, assuming that the proteins are undamaged during the polymerization. This amount is 650-fold less than the total applied amount of protein during polymerization. This amount of remaining protein on the nanocomposite is almost double the amount of Mb in comparison to Si@p(NIPA-*co*-APMA). In the random copolymerization approach, the product has 0.141 mg Mb/mg particle, which is 850-fold lower than the initial concentration of the biocatalyst in the SI-ATRPase reaction. The higher amount of Mb in the Si@pNIPA-*b*-pAPMA nanocomposite can be explained by the two-step ATRP polymerization and the use of the protein as a biocatalyst in each phase. In addition, the molar ratios between the monomers and the biocatalyst in the two approaches are different. In the random copolymerization, the molar ratio of NIPA/APMA/MB/AscA was 1:0.22:0.0016:0.06. The molar ratio of NIPA/Mb/AscA in the first step of block copolymerization is 1:8 × 10^–4^:0.033, and in the second step, the ratio of APMA/Mb/AscA is 1:0.03:0.09. Differences in the molar ratio of the monomers and the biocatalyst can have an impact on the formation of grafted polymer brushes as a consequence of their physical and chemical characteristics. Additional work to identify the optimum molar ratio arrangement is required to have a better understanding of the materials and to perform a rational comparison between the two nanocomposites.

The amount of iron on the nanocomposite decreased by over 90% (Table 1) after postmodification. This amount in Si@p(NIPA-*co*-APMA)_BA_ and Si@pNIPA-*b*-pAPMA_BA_ is hypothetically equal to 0.009 and 0.015 mg Mb/mg particles, respectively. Mb protein has a complex structure in comparison to inorganic catalysts. A small part of the protein acted as a catalyst during ATRPase and remained unreacted, while another part became covalently/noncovalently block the bromide.^25^ One simple explanation for the lower iron content after postmodification can be that those proteins connected covalently to the bromides have broken down or denatured to some extent under high alkaline conditions during postmodification. Therefore, the residues of other amino acids, as well as the iron center, can be washed off easily from the nanocomposite.

The hydrodynamic size of the particles measured by DLS is in keeping with the SEM images (Table 2). The differences can be linked to the nanocomposite states analyzed in the dry and wet states. The measurements were performed at room temperature and 40 °C (above LCST_NIPA_) to study the effect of the temperature on the grafted brush. The size of the Si@pNIPA nanoparticles decreased (16%) when the temperature increased due to the collapse of the pNIPA brushes. Over the LCST, pNIPA faces reversible dramatic chain dehydration and aggregates as the temperature increases.^28^ This thermoresponsive character of pNIPA indicates the possibility of controlling its chemistry and architecture as desired by changing the temperature. The grafted brush was swollen to its original size when the temperature decreased.

**Table 2:** Hydrodynamic Size and ζ Potential of the Particles Measured in Distilled Water

sample name	*T* (°C)	PdI	average size (nm)	ζ potential
silica nanoparticle	20	0.036	188.7 ± 5.6	–50.5 ± 0.7
	40	0.031	183.5 ± 2.3	–45.0 ± 0.5
Si@NH_2_	20	0.212	190.5 ± 10	5.12 ± 1.8
	40	0.234	194.7 ± 2.3	1.42 ± 1.1
Si@initiator	20	0.301	210.3 ± 3.0	28.4 ± 0.8
	40	0.159	212.5 ± 5.2	23.8 ± 2.5
Si@pNIPA	20	0.124	293.5 ± 1.5	–22.2 ± 1.1
	40	0.423	246.4 ± 2.4	–20.3 ± 0.9
Si@p(NIPA-*co*-APMA)	20	0.208	381.2 ± 6.8	5.7 ± 0.6
	40	0.155	409.6 ± 3.9	2.6 ± 0.7
Si@pNIPA-*b*-pAPMA	20	0.067	400.7 ± 7.2	–5.1 ± 0.1
	40	0.371	372.9 ± 5.5	–2.0 ± 0.9
Si@p(NIPA-*co*-APMA)_BA_	20	-	-	–34.6 ± 0.6
	40	-	-	–31.1 ± 4.4
Si@pNIPA-*b*-pAPMA_BA_	20	-	-	–51.3 ± 8.4
	40	-	-	–43.8 ± 6.3

However, the Si@p(NIPA-*co*-APMA) nanocomposite acted differently toward the temperature changes. It was expected that the size of the particles decreased at 40 °C,^24^ but it increased by 7%. It was reported that under ionic conditions, APMA has higher reactivity than NIPA (*r*_NIPA_ < 1 and *r*_APMA_ > 1), which will lead to propagation and a more random distribution of APMA units in the copolymer chain than NIPA units.^29^ This condition is the same in the random copolymerization via the SI-ATRPase reaction, which can result in more APMA units on the polymer brushes than NIPA. The numbers suggested that while the pNIPA chain becomes hydrophobic at higher temperatures, pAPMA with its cationic groups in the grafted brushes creates strong coulombic repulsion, which inhibits thermo-induced chain collapse.^30^ Thus, there is a struggle between the two building block units, and the 7% increase in the hydrodynamic size is the outcome of the competition between them.

The average size of the Si@pNIPA-*b*-pAPMA particles decreased, as expected, with increasing temperature. The first layer of the grafted brush, pNIPA, collapses and becomes more hydrophobic than the extended brush, pAPMA, when the temperature rises. In this approach, the cationic group of APMA units is located in the external layer of the polymer chain; hence, these units do not dramatically affect thermoresponsive changes of the pNIPA-grafted brush.

The changes on the surface of particles after each modification step can be followed by changes in ζ potential (Table 2). Introducing the amine group on the nanocomposites has a significant impact on the ζ potential and causes the particles to agglomerate.^31^ The temperature changes decreased the value of the ζ potential of the particles; however, it did not have a substantial impact on their charges (Table 2). The value of the ζ potential of both types of nanoparticles increased after introducing boronic acid ligand, which resulted in a relatively stable particle dispersion in an aqueous solution.

The organic content in the different particles, Si, Si@NH_2_, Si@initiator, Si@pNIPA, Si@pNIPA-*b*-pAPMA, and Si@p(NIPA-*co*-APMA), was assessed by thermogravimetric analysis (TGA) analysis (Figure S7). The TGA results revealed an ∼1.6 wt % difference in weight retention at 750 °C between bare silica and Si@NH_2_ particles, which is attributed to modification with APTES (curves a and b). The difference in weight loss between Si@NH_2_ and Si@initiator particles is ∼0.48 wt %, which confirms the immobilization of the initiator molecules (curve c). The amount of initiator immobilized on silica is ∼0.18 mmol g^–1^ (using the mass retention of Si@initiator at 750 °C as a reference). For Si@pNIPA, Si@pNIPA-*b*-pAPMA, and Si@p(NIPA-*co*-APMA) particles, a larger weight loss occurred when the temperature increased from 250 to 750 °C due to the decomposition of the organic polymer on the surface. The amounts of NIPA and APMA grafted on silica via block polymerization are calculated to be ∼0.5 and 0.3 mmol/g silica, respectively (curves d and f). For the Si@pNIPA-*b*-pAPMA particles, the molar ratio of NIPA to APMA in the block copolymer brushes was calculated to be approximately 1.6:1. However, blockage of some of the initiator’s sites during the ATRPase process by the biocatalyst has an impact on the grafted polymer chain compared to the traditional ATRP process.^24^ The amount of organic chemicals on Si@p(NIPA-*co*-APMA) composite particles was calculated to be ∼0.091 g/g silica, which is slightly higher than that on Si@pNIPA particles. It should be noted that the amount of Mb residual has not been considered in the above calculation. The mass retentions of the Si@pNIPA-*b*-pAPMA_BA_ and Si@p(NIPA-*co*-APMA)_BA_ particles were calculated to be 18.1 and 12.0%, respectively, which is the result of postmodification by epichlorohydrin and phenylboronic acid (curves g and h).

To determine the molecular weight of the grafted polymer brushes on Si@pNIPA, the nanoparticles were treated with HF solution to etch the silica core. Unfortunately, the extracted pNIPA was difficult to dissolve in dimethylformamide (DMF) or other common organic solvents (Figure S8). Thus, it was not possible to analyze the sample with gel permeation chromatography (GPC). The poor solubility may be explained by the presence of Mb residuals in the extracted polymer. For the same reason, Si@pNIPA-*b*-pAPMA and Si@p(NIPA-*co*-APMA) cannot be analyzed by GPC.

### Boronate Affinity Adsorbents

Using Mb as a catalyst in the ATRP reaction has benefits, such as the possibility of applying the final product in biomedical and drug delivery applications. However, blocking the initiator by Mb has a negative impact on the rate of the reaction, and consequently, there is a low yield of postmodification. Thus, having an additional functional group on the grafted brush can address this challenge. To demonstrate a bioseparation application for the core–shell nanocomposite, a batch binding adsorption experiment was designed. The amine groups on the APMA monomer were utilized to attach PBA to both types of materials and form boronate affinity adsorbents. It is possible to tailor these core–shell nanocomposites with other ligands to create adsorbents, such as immobilized metal ion affinity, dye affinity, and 5-thio-2-nitrobenzoic acid (TNB)-thiol affinity ligands.^32^

To proceed with the postmodification, first, an epoxy ring was coupled to the amine group, and then the phenylboronic acid ligand covalently bound to the grafted brush via the pendant epoxy group. The presence of the ligand was confirmed by elemental analysis (Table 1). This type of adsorbent can be used for the purification of a wide range of macromolecules, such as glycoproteins, carbohydrates, nucleic acids, and polyphenols.^33^ The reversible esterification reaction between the PBA ligand and diols/sugars of macromolecules is the key for the selectivity of the ligand^34,35^ and has been used extensively in many studies.^36,37^ The positioning of the boronic acid and the hydroxyl groups of the adsorbate are the main factors in the primary interaction.^34^ In comparison to lectins and other biobased ligands, this adsorbent has a longer shelf life and is robust. In addition, phenylboronic acid can be operated under harsh conditions, e.g., at high/low pH and temperatures.^33^

**Figure 5:**
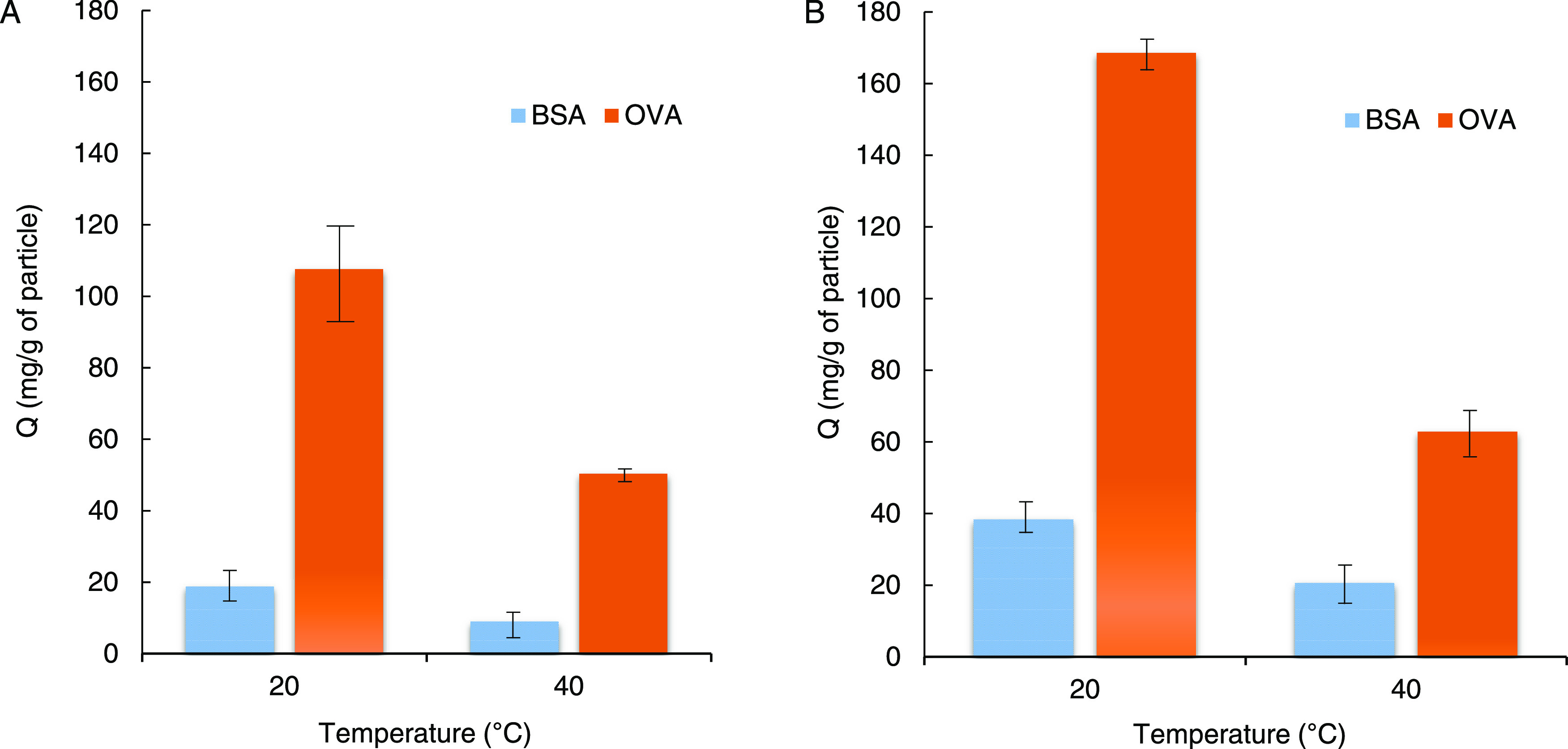
Effect of temperature on the binding of BSA and OVA to (A) Si@p(NIPA-*co*-APMA)_BA_ and (B) Si@pNIPA-*b*-pAPMA_BA_ nanocomposites.

Three different types of proteins (BSA, OVA, and HbA1c) were selected to study the boronate affinity particles. Glycated hemoglobin is used as a diagnostic test for diabetes, where glucose can attach to the N-terminal residue of the β subunit in HbA1c.^38^ OVA, a well-known glycoprotein, is the main protein in egg white and has a molecular mass of 42 kDa, and its isoelectric point (pI) is 5.19.^39^ BSA (66.5 kDa and pI 4.7) is a nonglycated protein and was used as a control. The absorption of each protein was measured by a UV/vis spectrophotometer at wavelengths of 280 nm (BSA and OVA) and 577 nm (HbA1c). The concentrations of OVA and BSA were calculated using the molar extinction coefficients ε_280_ = 4.2 × 10^4^ and 6.6 × 10^4^ M^–1^ cm^–1^, respectively. The molar extinction coefficient of HbA1c was calculated as ε_577_ = 7.7 × 10^3^ mM^–1^ cm^–1^. The concentration of protein was followed during the adsorption process (Figure S9). NaCl in running buffer minimizes the electrostatic interaction between the proteins and negatively charged PBA. The binding capacity of the Si@p(NIPA-*co*-APMA)_BA_ nanocomposite (based on eq 1) toward BSA and OVA at 20 °C were 18 and 107 mg/g particles, respectively (Figure 5A). The binding capacity of the block copolymerization nanoparticle Si@pNIPA-*b*-pAPMA_BA_ at 20 °C was calculated to be 38 and 163 mg/g particles for BSA and OVA, respectively (Figure 5B). The selective binding capacity toward HbA1c at 20 °C was calculated as 151 and 209 mg/g particles for random copolymer nanocomposite and block copolymer nanoparticles, respectively. In Si@(pNIPA-*b*-pAPMA)_BA_ particles, the ligand is immobilized on the second layer of the brush (the extended chain), which is different from the Si@p(NIPA-*co*-APMA)_BA_ nanocomposite where the ligand is randomly distributed alongside the grafted chains (Figure 1). The distribution pattern of the ligand affects the accessibility of the proteins to the adsorbent and thus the binding capacity of the produced material. Under the mentioned operational conditions, both materials revealed a low capacity toward the control protein BSA. The short period of the washing process might not be sufficient to remove all of the proteins bound nonspecifically to the polymer brushes. Hence, a longer washing time is required. However, the eluted amount of BSA relative to the amount of adsorbed glycated proteins (OVA and HbA1c) can be overlooked. It should be noted that the same batch of particles could be regenerated and reused repeatedly.

The theoretical capacity of the particles for adsorbing OVA, based on the boron concentration (Table 1), is approximately 18- and 30-fold greater for the Si@p(NIPA-*co*-APMA)_BA_ and Si@pNIPA-*b*-pAPMA_BA_ nanocomposites, respectively. This large gap between the theoretical and achieved results can be explained by the size of the adsorbate and the fact that the target proteins do not have access to the ligands.

By increasing the temperature to 40 °C (above LCST_NIPA_), the binding capacity of the nanocomposite decreases. This finding can be explained by reducing the distance between the side chains on the grafted brush because of the collapsing pNIPA. As mentioned earlier, there are more APMA units than NIPA in the polymer chains of Si@p(NIPA-*co*-APMA)_BA_, but the accessibility of the macromolecules to the pendant PBA decreases as a result of partially collapsing brushes, and the binding capacity of the adsorbents diminishes. In fact, the two nanocomposite binding capacities at 40 °C are close to each other under the mentioned operating conditions. Conducting the experiment for glycated hemoglobin at 40 °C cannot be reported since HbA1c is unstable at this temperature.

Sodium dodecyl sulfate polyacrylamide gel electrophoresis (SDS-PAGE) analysis was conducted to evaluate the quality of the elution fractions for the three proteins at different temperatures (Figure S10). Although the collected samples were concentrated, no visible band at 67 kDa could be detected for BSA in the elution fractions at either 20 or 40 °C (Figure S10A,B, lanes 3 and 5). The bands related to OVA and HbA1c appeared at 42 and 16 kDa, respectively (Figure S10A,B, lines 7, 9, and 11). Since all of the loaded samples for SDS-PAGE were concentrated before the analysis, the strengths of the bands cannot be correlated to the protein concentrations in their respective fractions.

### Selectivity toward Mb

The physical and chemical changes in the structure and morphology of the nanocomposite can be followed by SEM after postmodification. Figure S11B,C illustrates that individual nanocomposite particles aggregate to some extent and form larger particles. At a high magnification, the SEM images reveal that the surface of the nanocomposite becomes rougher after postmodification (Figure S11b,c), which can be a result of the cross-linking on the polymer brushes. Although the SEM image of the modified core–shell particles with boronic acid (Figure S11c) revealed some particle–particle aggregation (in a dried state), the particle suspension shows an overall stable colloidal suspension in water (ζ potential, Table 2), which makes it possible for the proteins to have access to the polymer brushes. Even applying a high concentration of epichlorohydrin did not stop some of the epoxy groups from reacting with free amine groups on the polymer chains and causing cross-linking within the polymer brushes and with the neighboring core–shell particle. The same effect was observed in SEM images of Si@pNIPA-*b*-pAPMA_BA_ (data not presented). It should be noted that modification with epichlorohydrin is only an example to exhibit an application for the nanocomposite. Other approaches for introducing ligands or functional groups can be conducted to avoid cross-linking and side reactions.^32^

Utilizing Mb as a catalyst in the ATRPase reaction shows that the product cannot be free of the catalyst even after intensive washing with different organic solvents and water. This finding can be explained by the reaction between residual histidines on Mb and the initiator.^25^ The core–shell nanocomposites are dark red after polymerization, and the color fades during postmodification into light beige and stays in that state (Figure S12). The reddish color is an indication of the presence of iron on the nanocomposite, which can be used to determine the theoretical amount of Mb after the ATRPase reaction on the particles (reported earlier). During the postmodification procedure, these remaining biomolecules can act as a template, where epichlorohydrin cross-links the polymer brushes. The reaction situation evokes the molecular imprinting technique to form MIP particles. Molecularly imprinted polymer (MIP) particles are prepared by cross-linking functional monomers in the presence of a target (bio) molecule known as a template. By removing the target molecules after polymerization, there will be cavities on the surface of the material that mimic the specific shape, size, and functional groups of the template and enable highly selective molecular recognition toward the target.^40−42^ This unintended alternation of the nanocomposites displays selectivity toward Mb. A similar approach was used by Sun’s group to develop sensors by applying ATRPase.^17,18^

To demonstrate the selectivity of the particles, a mixture of Mb with a competitive analog protein, BSA, was used. After incubating the core–shell nanostructure in a solution of Mb and the interfering protein, the particles were washed to remove the nonspecific binding proteins, and then the target molecules were eluted. The elution fractions were analyzed by spectrophotometry and SDS-PAGE. The molar extinction coefficient of Mb was calculated as ε_409_ = 2.3 × 10^3^ M^–1^ cm^–1^. The concentration of Mb in each fraction during the adsorption/elution procedure is reported in Figure S13.

**Figure 6:**
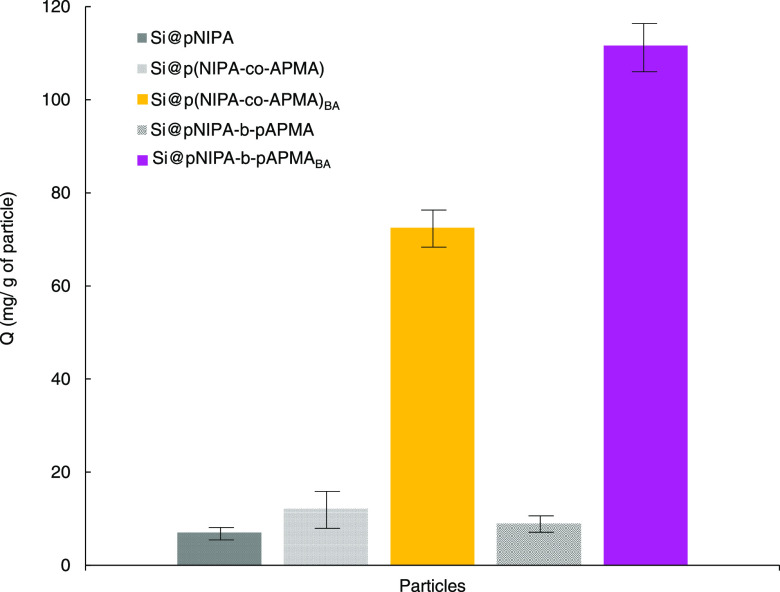
Selectivity of the nanocomposite toward Mb before and after postmodification.

Using the Mb concentration in the elution fraction and eq 1, the capacity of the designed nanocomposite was calculated to be 72 and 111 mg/g for Si@p(NIPA-*co*-APMA)_BA_ and Si@pNIPA-*b*-pAPMA_BA_, respectively (Figure 6). Both NIPA and APMA are acrylamide-based monomers. Their selectivities and affinities toward different proteins, such as Mb, for developing MIP particles have been reported elsewhere.^43^ In the block copolymer approach, there are more distinctive brushes around the core. Moreover, the presence of more Mb on the material can increase the imprinting impact. Thus, the binding capacity of Si@pNIPA-*b*-pAPMA_BA_ is higher than that of Si@p(NIPA-copAPMA)_BA_. The cross-linker agent and the postmodification process play an essential role in forming so-called MIP particles in this experiment. Hence, an imprinting effect cannot be seen on the nanocomposite before postmodification (Figure 6). The amount of Mb eluted from particles before modification was very low in comparison to the modified nanocomposite. This amount can be reduced even more if the washing step is completed under a more extended period.

SDS-PAGE analysis was conducted to examine the presence of BSA in the elution fraction (Figure S14). Mb and BSA bands appear at 17 and 67 kDa, respectively. It is clear that the adsorbents were able to separate Mb even in the presence of BSA (Figure S14, lanes 3 and 5), which indicates a higher selectivity of the cross-linked brushes on the core–shell nanocomposite toward Mb. The appearance of a weak band at approximately 50 kDa (Figure S14, lane 5) could be related to BSA protein. Although the band is very weak, two scenarios can be described: (1) short and inefficient washing steps and (2) imprinting operational conditions. In the case of the first assumption, a more extended washing period is required to remove the trapped proteins from the cross-linked network. At this stage, it is crucial to plan a more detailed study on this approach to fully understand the imprinting factor. However, a simple explanation can be that myoglobin broke down in a highly alkaline condition into a cocktail of polypeptides where each of them can participate in the pseudoimprinting process at the same time. As a result, not all of the artificial affinity cavities on the polymer brushes can be an accurate footprint of the Mb protein. In this situation, it could be fair to assume that some of the affinity sites could show selectivity toward competitor molecules such as BSA. Nevertheless, these findings can offer a new approach for the formation of MIP nanoparticles toward large (bio)molecules, such as hemoglobin. By optimizing the operational conditions, this method can provide a larger surface area with smaller particle size and narrower size distribution. Other available techniques, such as Pickering emulation polymerization,^44^ cannot provide such features. The presence of both proteins can be observed in the elution fractions in the SDS-PAGE analysis of the particles before postmodification (Figure S15). These results are expected since there is no ligand or functional group on the nanocomposite that has an affinity toward either Mb or BSA.

To study the behavior of the Si@p(NIPA-*co*-APMA)_BA_ and Si@pNIPA-*b*-PAPMA_BA_ nanocomposites as bifunctional materials, the two particles were exposed to a mixture of Mb and OVA. The affinity of the particles toward the two proteins was assessed at different pH values (6, 7.5 and 9). The preliminary plan was to have a qualitative rather than quantitative analysis using SDS-PAGE. This analysis was performed via a comparison of the intensity of the protein bands among the unbound fractions (supernatants). The sample fractions were not concentrated, and they were applied to the gel as it was collected. The bands of the original loading mixture appear to not be stretched, as in the other lanes (Figure S16, lane 1). This finding can be explained by the field effect and unevenly distributed current during the process where the spacers interrupt the electric field at the edges of the gel. At pH 9 (above phenylboronic acid p*K*_a_), PBA will shift its equilibrium toward a tetragonal configuration, which favors *cis*-diol binding.^33^ Therefore, more glycoprotein binds to the ligands on the particles and fewer proteins remain in the supernatant. Consequently, the band of OVA from the supernatant appears weaker than that of the other pH values (Figure S16, lanes 3 and 7). At the same pH, the particles have lower affinity adsorption toward Mb. The adsorption of Mb based on the imprinting effect occurs at pH 6 (below Mb pI: 6.8), and the elution process occurs at a higher pH (pH 9). Therefore, less Mb interacts with the nanocomposite at a higher pH and more can be found in the supernatant (stronger band on SDS-PAGE, Figure S16, lanes 3 and 7). The Mb band on SDS-PAGE appears to be weaker at pH 6 (more proteins were adsorbed to the particles) than at pH 9.

At low pH (below the p*K*_a_ value of PBA), the reaction will shift the equilibrium toward boronate ions (trigonal confirmation), which does not bind *cis*-diol. Therefore, less OVA was attached to the phenylboronic ligand at pH 6 (Figure S16, lanes 1 and 5). The main competition might be at approximately pH 7.5, where the nanocomposites could show double functionalities. These preliminary results only indicate the different behaviors of the nanocomposite at various pH values. More detailed studies have already been scheduled in our pipeline for more quantitative analysis.

## Materials and Methods

### Materials

The following were obtained from Sigma-Aldrich (Sweden) and used as received unless otherwise stated: NIPA, 3-aminophenyl boronic acid hemisulfate salt (≥95%) (APBA), APMA, methanol, ammonia solution 25%, tetrahydrofuran (THF), tetraethylorthosilicate (TEOS), (3-aminopropyl)triethoxysilane (APTES), potassium ferricyanide, hydrofluoric acid (40%), potassium cyanide, Tween 20, toluene, acetic acid, triethylamine, 2-bromoisobutyryl bromide (BIBB), 2-hydroxyethyl 2-bromoisobutyrate (HEBIB), acetone, sodium chloride (NaCl), sodium hydrogen phosphate, sodium dihydrogen phosphate, sodium carbonate, ascorbic acid (AscA), epichlorohydrin, myoglobin from equine skeletal muscle (Mb), OVA, and BSA. Sodium hydrogen carbonate (NaHCO_3_, 99%) was obtained from Merck (Germany). HbA1c was obtained from Bio-Rad Laboratories (China). The sodium dodecyl sulfate polyacrylamide gel electrophoresis (SDS-PAGE) kit was purchased from Bio-Rad Laboratories, Sweden.

### Synthesis of SiO_2_ Nanoparticles

Silica nanoparticles were prepared as described elsewhere^44^ using a one-step Stöber procedure, where water (33 mL), methanol (100 mL), and ammonia (25%) (22.4 mL) were added to a round bottom flask. TEOS (13.8 mL) was mixed with methanol (130 mL) and added to the mixture. The solution was stirred at room temperature for 8 h. The formed nanoparticles were isolated by centrifugation at 8000 g, washed with methanol and water (three times), and dried in a vacuum desiccator overnight at room temperature.

### Preparation of Amino-Functionalized Silica Nanoparticles (Si@NH_2_)

The produced silica nanoparticles (3.0 g) were added to 1% APTES solution (in anhydrous toluene) and stirred for 24 h at reflux temperature (110 °C). The particles were isolated using centrifugation and washed three times, first with acetone and then with methanol. The washing process was continued with water (two times), and then the particles were dried in a vacuum desiccator at ambient temperature overnight. The product is denoted as Si@NH_2_.

### Introduction of Initiator-Functionalized Si@ NH_2_ (Si@Initiator)

The ATRP initiator was immobilized on Si@NH_2_ using a procedure described in our previous work.^24^ Si@NH_2_ (250 mg), triethylamine (0.4 mL), and THF (12 mL) were mixed and stirred in a glass flask in an ice-water bath. BIBB (0.62 mL) was added to the mixture dropwise. Then, the reaction mixture was removed from the ice bath and stirred at room temperature overnight. After collecting the nanoparticles by centrifugation, methanol was used to wash the particles (three times) on a rocking table at room temperature. Then, the solvent was replaced by distilled water, and the procedure was repeated. Finally, the particles were dried at room temperature under vacuum and denoted as Si@initiator.

### Synthesis of pNIPA Brushes Grafted from Silica Nanoparticles (Si@pNIPA)

Si@initiator nanoparticles (100 mg) and NIPA (1040 mg) were added to 15 mL of water. The suspension was sonicated for 5 min and then stirred and degassed by purging nitrogen. Mb (28 mg/mL, 4.3 mL) was degassed by purging N_2_ gas for a minimum of 30 min and then sealed. AscA (64 mg) was dissolved in 3 mL of water, degassed under N_2_ gas, and sealed. To the silica nanoparticle suspension, AscA solution (2 mL) was added while the mixture was purged with N_2_ gas. Then, the Mb solution was added under the same conditions. The reaction bottle was then closed, and the reaction continued for 24 h at room temperature. After the reaction time, the particles were centrifuged (8000*g*) and then washed with ethanol and water (three times). The core–shell nanocomposite was dried under vacuum at room temperature and denoted Si@pNIPA.

### Synthesis of Free pNIPA Chain (Control Experiment)

NIPA (346 mg) and HEBIB (4.3 μL) were dissolved in 5 mL water and degassed by purging nitrogen. AscA solution (10 mg/mL, 0.66 mL) was prepared and degassed separately. Mb solution (14 mg/mL, 1.3 mL) was degassed under nitrogen gas for approximately 20 min. The two solutions were added to the monomer mixture, degassed for 10 min, and sealed. The reaction was allowed to stir at room temperature for 24 h. For the control experiment, Mb was dissolved in Drabkin solution^45^ instead of water, and the reaction procedure was followed under the same conditions as mentioned.

### Introducing Block Copolymer Brushes Grafted from Silica Nanoparticles (Si@pNIPA-*b*-pAPMA)

Si@pNIPA nanoparticles (25 mg) and APMA monomer (50 mg) were suspended in water (2.6 mL) and sonicated for 10 min. The suspension was degassed by purging nitrogen. Mb solution (28 mg/mL, 2 mL) was prepared and degassed by purging N_2_ and sealed. AscA (21 mg/mL, 1 mL) was degassed using nitrogen gas and added to the suspension under N_2_ gas. Mb solution (1.4 mL) was added to the reaction mixture. Purging with N_2_ gas was continued for another 10 min, and then the bottle was sealed. The reaction was stirred for 24 h at room temperature. The particles were separated by centrifugation at 8000*g*. Then, they were washed with ethanol (three times) and water (three times) and dried at room temperature under a vacuum desiccator. The nanocomposite was denoted as Si@pNIPA-*b*-pAPMA.

### Preparation of Random Copolymer Brushes Grafted from Silica Nanoparticles (Si@p(NIPA-*co*-APMA))

Si@initiator nanoparticles (100 mg), NIPA (600 mg) and APMA (200 mg) were added to water (15 mL). The mixture was sonicated for 5 min and then stirred and degassed by purging nitrogen. The reaction procedure was followed as described earlier for Si@pNIPA. The nanocomposite was denoted as Si@p(NIPA-*co*-APMA).

### Preparation of Core–Shell Nanocomposite Containing Pendant Epoxy Group

Si@pNIPA-*b*-pAPMA (30 mg) was suspended in a mixture of water and 2 M NaOH (5 mL, v/v: 1/1). The suspension was sonicated for 5 min, and then epichlorohydrin (3 mL) was added to the mixture. The reaction was stirred overnight at 40 °C. The separation procedure was started with the centrifugation of particles at 8000*g* and continued with washing with methanol (three times). Then, the solvent was replaced by water, and the procedure was repeated. Finally, the nanocomposite was dried at room temperature under a vacuum desiccator and denoted Si@pNIPA-*b*-pAPMA_ep_.

The same procedure was followed to introduce a pendant epoxy group on Si@p(NIPA-*co*-APMA) particles. The nanocomposite was denoted as Si@p(NIPA-*co*-APMA)_ep_.

### Introducing Boronic Acid Ligand on Core–Shell Nanocomposite

Si@pNIPA-*b*-pAPMA_ep_ (15 mg) was suspended in 0.1 M carbonate buffer pH 9 (5 mL) using a sonication bath. APBA (15 mg) was added to the suspension. The reaction was stirred at room temperature for 24 h. The silica nanoparticles were collected by centrifugation, washed with methanol (three times) followed by water (three times), and dried at room temperature under vacuum. The nanocomposite was denoted as Si@pNIPA-*b*-pAPMA_BA_.

Si@p(NIPA-*co*-APMA)_ep_ reacted under the same conditions as the APBA ligand, and the nanomaterial was denoted as Si@p(NIPA-*co*-APMA)_BA_.

### Protein Binding on Si@p(NIPA-*co*-APMA)_BA_ and Si@pNIPA-*b*-pAPMA_BA_ Nanocomposites Based on Boronic Ligands

Both materials were evaluated for batch adsorption separately at two different temperatures, 20 and 40 °C. BSA (2 mg/mL), OVA (2 mg/mL) and HbA1c (5 mg/mL) were selected for the adsorption experiment. The experimental conditions were kept the same for all reactions.

Silica-grafted nanocomposite (2 mg) was suspended in running buffer (1 mL) (0.1 M carbonate buffer pH 9.0 containing 0.1 M NaCl). The proteins were added separately to each container based on the mentioned concentration. The reaction tubes were placed on a rocking table for 24 h at the chosen temperature.

After the reaction was completed, the particles were centrifuged, and the supernatant was collected and read by UV/vis spectrophotometer at 280 nm (for BSA and OVA) and 577 nm (for HbA1c). The particles were washed for 1 h at room temperature with running buffer (2 mL) to remove the nonspecifically bound proteins. The fractions were collected after centrifugation and measured by a spectrophotometer. Then, 0.1 M fructose in running buffer (1 mL) was added to the particles and placed on a rocking table for 24 h to elute the protein from the boronic acid ligand. The elution fraction was collected and read by a spectrophotometer.

The capacity of the particles was calculated using the equation below
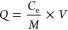
1where *Q* is the bound protein per unit mass (mg/g of particle), *C*_e_ (mg/mL) is the concentration of the protein in the elution fraction, *M* (g) is the mass of the adsorbent (nanocomposite), and *V* (mL) is the volume of the protein solution.

The collected fractions were freeze-dried and analyzed by SDS-PAGE.

### Selectivity Test toward Mb

The selectivity of the nanocomposite before and after postmodification was assessed in a mixture of Mb (2 mg/mL) and BSA (4.1 mg/mL). The adsorption/elution process was performed as described above, except that phosphate buffer (0.1 M, pH 6.0, 1 mL) was used as a running buffer, and carbonate buffer (0.1 M, pH 9.0, 1 mL) was used to elute the bound Mb from the nanocomposite. After each step (loading, washing, and elution), the nanocomposite was centrifuged. The supernatant was collected and read by a UV/vis spectrophotometer at 409 nm wavelength, and the fraction from each step was collected for SDS-PAGE analysis.

The postmodified nanocomposites (2 mg) were added to a mixture of Mb and OVA (1 mL) at different pH values (6.0, 7.5, and 9.0) to study their behavior toward Mb and a glycoprotein at the same time. The mixture was incubated with the particles at room temperature for 24 h. The initial concentration of the proteins was 5 mg/mL. The particles were centrifuged, and the supernatants were collected for SDS-PAGE analysis.

### Regenerate the Silica-Grafted Nanostructure

The nanocomposite was washed with a 10 mM acetic acid solution for 30 min after each adsorption/elution procedure to remove any remaining protein. Then, they were washed with water until the pH of the supernatant became neutral and finally dried under vacuum at ambient temperature.

### Characterization

A Nano-ZS 3600 particle sizer (Malvern Instruments, Malvern, U.K.) was used to determine the ζ potential, mean size, and particle size distribution of the silica nanoparticles. Fourier transform infrared (FTIR) spectroscopy (Nicolet iS5, Thermo-Fisher Scientific, Inc., Waltham, MA) was used to study the chemical changes after each synthetic step, with a resolution of 4 cm^–1^ and 16 scans.

The structure of the materials was studied using a scanning electron microscope (SEM), a JSM-6700F electron microscope (Oxford, U.K.), and a transmission electron microscope (TEM) equipped with a field-emission gun (JEOL, model 3000F, Japan). For SEM imaging, a thin layer of gold (∼15 nm) was sputtered onto the surface of the dried samples. The operational conditions were 10 kV with a beam current of 10 μA.

Elemental analysis (C, H, N, Br, Fe, and B) was performed by Mikroanalytisches Laboratorium Kolbe (Germany) using the following determination procedures. CHN-Analyzer-Vario Mikro Cube (Elementar, Germany) with a detection limit of 0.01% (determination error ±0.015%) was used to measure C, H, and N. Bromine was measured with an IC-883 Plus (Metrohm, Switzerland). The detection limit of the instrument was 0.005%, with a determination error of ±0.005%. Iron was analyzed with an AAS-Analyst 200 (PerkinElmer), with a detection limit of 50 ppm and a determination error of ±5 ppm. Boron was determined using a sensitive UV/vis spectrophotometer Analytik Jena-Specord 50 Plus (Endress+Hauser Company, Germany), with a determination error of 0.0005%.

Elemental mapping of the particles was performed by environmental SEM (E-SEM), with a tungsten filament with integrated electron backscatter diffraction (EBSD) and energy-dispersive X-ray (EDX) spectroscopy analytical systems. The instrument is an FEI Quanta MKII with a W filament (Eindhoven, the Netherlands), and the analytical EDX system has a polymer capillary window of 60 mm in diameter (energy-dispersive X-ray analysis (EDAX)). The accelerating voltage was set at 10 keV. The specimens were mounted on carbon tape with no additional sputtering layer.

UV–vis absorption spectra were recorded with a UV–vis spectrophotometer (Biowave II, Biochrome, U.K.). ImageJ 1.52a (Wayne Rasband, National Institutes of Health) and ChemDraw professional (PerkinElmer Informatics, Inc.) software were utilized as needed during the analysis and illustration.

Thermal gravimetric analysis (TGA) was carried out in synthetic air. The samples were heated at a rate of 10 °C/min. To measure the molecular weight of the polymer brush, Si@pNIPA composite particles (800 mg) were treated with 5% HF solution (25 mL) overnight to etch the silica core. The solution was then dialyzed against water for 2 days. The sample was collected and freeze-dried to measure the molecular weight by gel permeation chromatography (GPC) on a Viscothek GPCmax instrument equipped with a PFG column (300 × 8 mm^2^, 5 μm particle size) and a refractive index detector from PSS (Mainz, Germany). This analytical system uses dimethylformamide (DMF) containing LiBr (10 mM) as the mobile phase at a flow rate of 0.75 mL/min at 60 °C and polystyrene as standards for molecular weight calculation.

## Conclusions

Using heavy metals in the conventional SI-ATRP reaction as catalysts, even at low concentrations, is not environmentally friendly. In addition, their presence in the final product limits the application fields. Using a biomolecule in the “grafting from” approach can open up opportunities for core–shell nanostructures to be applied in more sensitive and complex areas, such as drug delivery and medical fields.

A thermoresponsive material based on a silica core was synthesized via SI-ATRPase in this work. Two different approaches, random copolymerization and sequential block copolymerization, were successfully implemented to design soft polymer brushes on a hardcore using water-soluble monomers. The addition of a functional group on the polymer brushes gives the flexibility to alter the material for final use. Customized nanocomposites were applied as boronate affinity ligands in this study. The fact that the materials were transformed into MIP nanoparticles during the modification process adds a new dimension to their functionalities. This work was only the beginning of exploring the SI-ATRPase reaction, and more research must be performed to understand the full mechanism of the polymerization as well as to optimize the properties of the polymer brushes and the effective control of each layer based on the desired requirements.

## Supporting Information Available

Additional results of material characterization and protein adsorption; the data include FTIR, TGA, SEM EDX, and SDS-PAGE of protein samples (PDF)
