# Supplementary material for: Synthesizing a Hybrid Nanocomposite as an Affinity Adsorbent through Surface-Initiated Atom Transfer Radical Polymerization Catalyzed by Myoglobin
Source: ACS Omega. 2021 Apr 12;6(15):10462–74. doi: 10.1021/acsomega.1c00955 (PMC8153740; doi:10.1021/acsomega.1c00955)
Supplement: Supplementary file 1 — ao1c00955_si_001.pdf [file ao1c00955_si_001.pdf]

Supporting information

Synthesizing hybrid nanocomposite as an affinity adsorbent through surface-initiated atom transfer radical polymerization catalyzed by myoglobin

Solmaz Hajizadeh, Leif Bülow, Lei Ye*

Division of Pure and Applied Biochemistry, Department of Chemistry, Lund University, 22100, Lund, Sweden

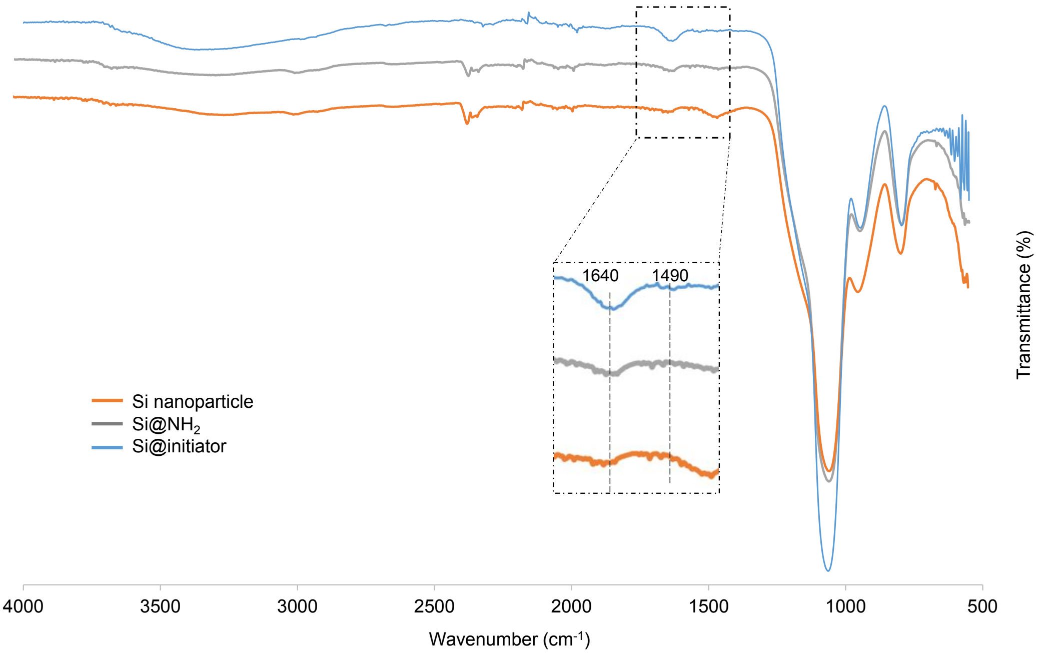

Figure S1. FTIR spectra of silica nanoparticles, Si@NH_2 and Si@initiator

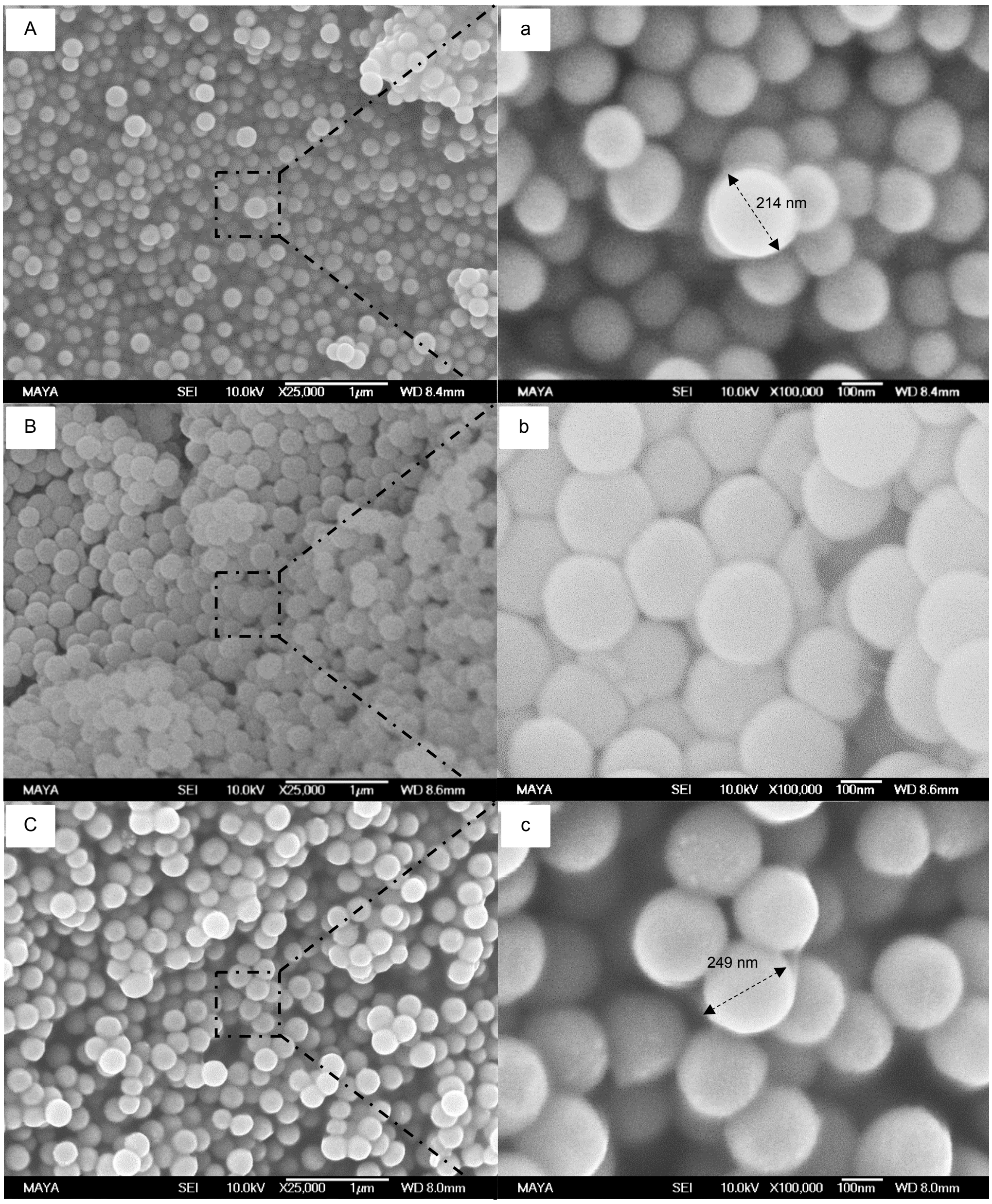

Figure S2. SEM images of A) silica nanoparticles; B) Si@NH_2 nanoparticles; C) Si@initiator nanoparticles (scale bar is 1 μm). Images a, b and c are the higher magnification of A, B and C, respectively with a scale bar of 100 nm. The arrows in images a and c, show the diameter of the particles before and after modification, respectively.

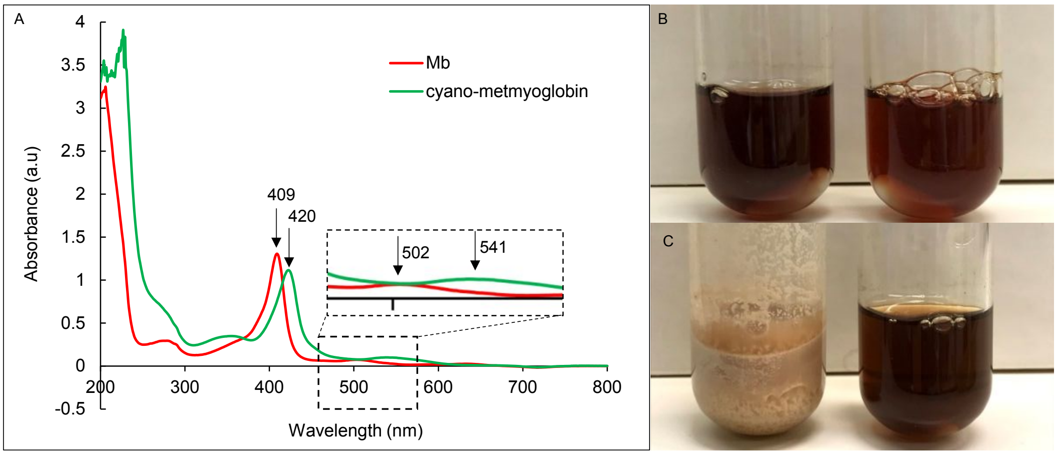

Figure S3. A) Spectra of the Mb before and after exposing to cyanide. Digital images of the completed ATRPase reaction at B) room temperature; C) 40 °C. In both images, the left bottle was catalyzed by commercial Mb, and the right bottle with cyano metmyoglobin.

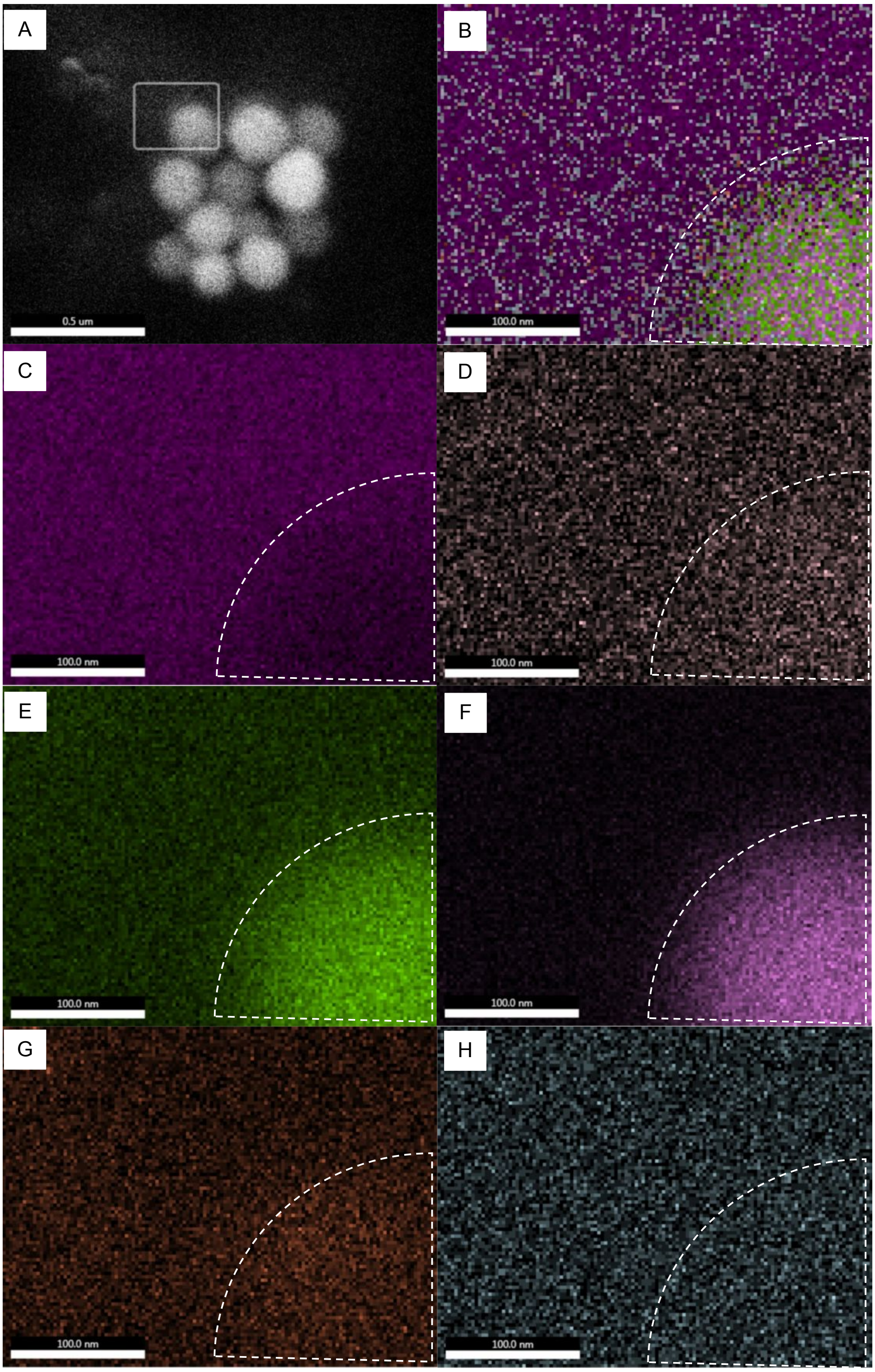

Figure S4. A) SEM image of Si@p(NIPA-co-APMA) nanocomposite with a scale bar of 500 nm. The white square is the selected area for the elemental mapping analysis. (B) Overlapped EDS-based elemental mapping; Mapping of the individual elements C) Carbon; D) Nitrogen; E) Oxygen; F) Silica; G) Iron; H) Bromide. The scale bar is

100 nm. The dashed white lines show the location of the particle in each image based on the elemental mapping of silica.

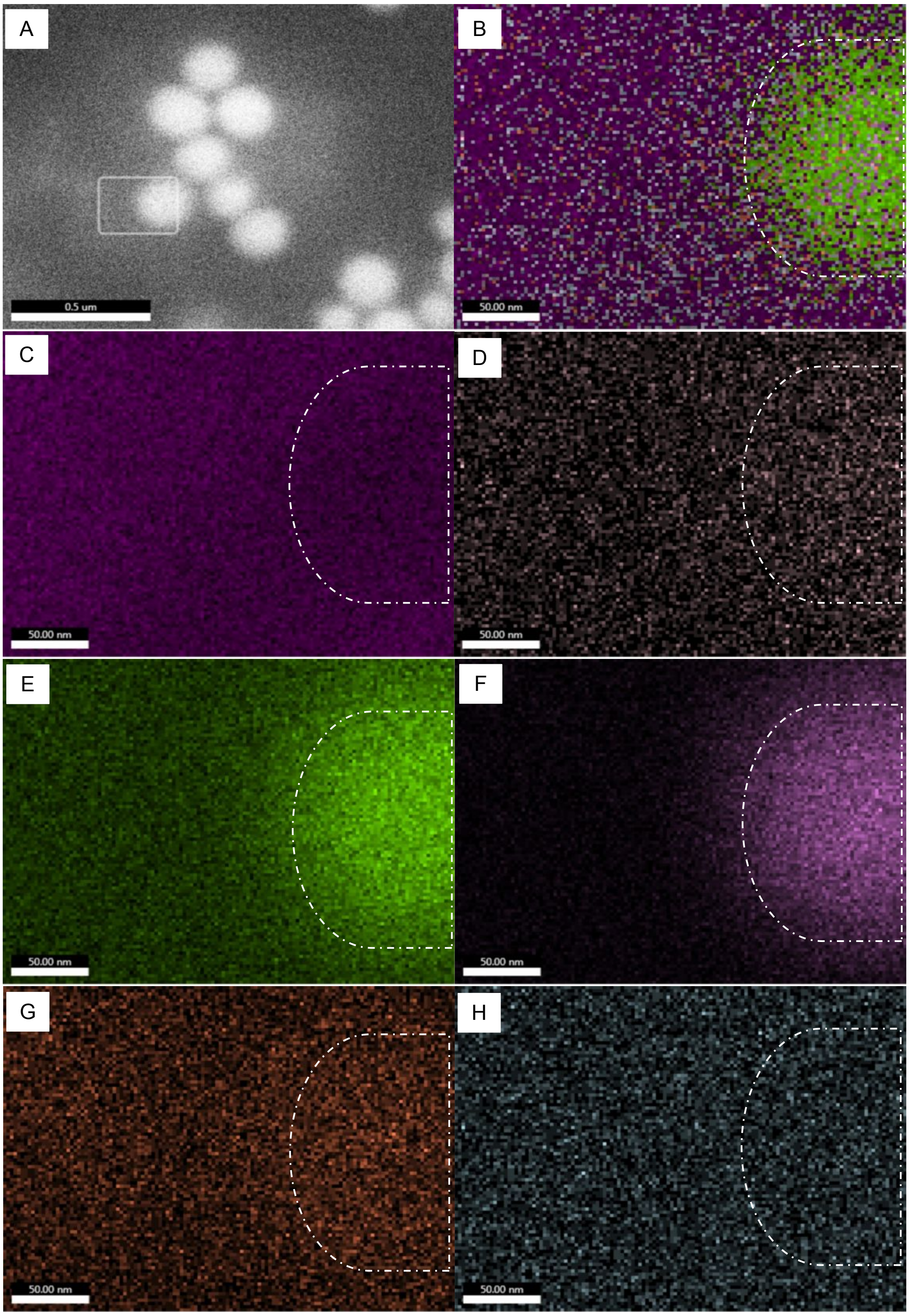

Figure S5. A) SEM image of the Si@pNIPA-b-pAPMA nanocomposite with a scale bar of 500 nm. The white square is the selected area for elemental mapping analysis. (B) Overlapped EDS-based elemental mapping; C) Carbon; D) Nitrogen; E) Oxygen; F) Silica; G) Iron; H) Bromide. The scale bars are 50 nm. The dashed white lines show the location of the particle in each image based on the elemental mapping of silica.

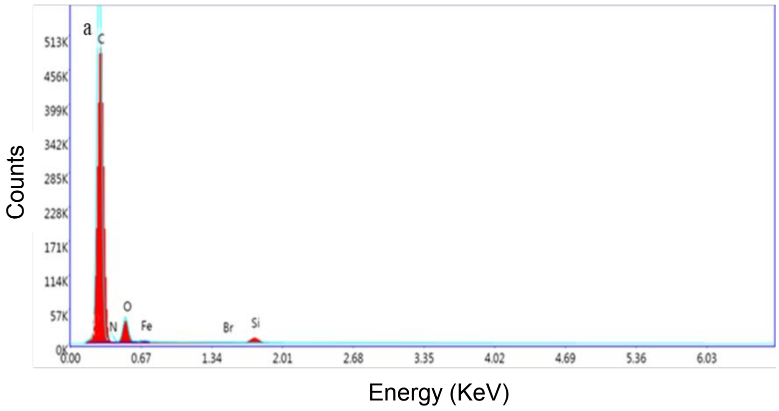

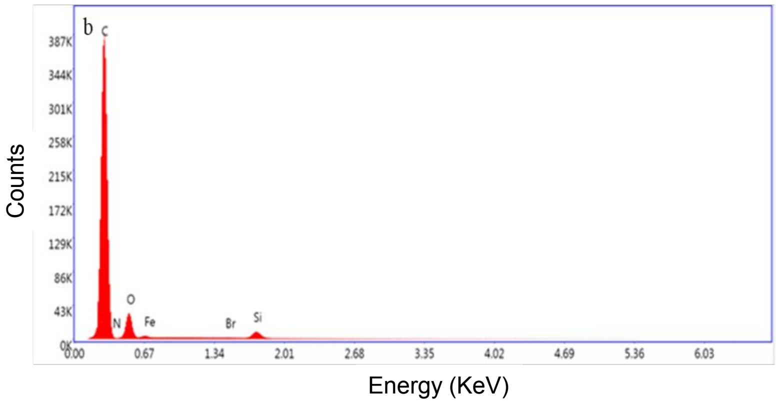

Figure S6. EDX spectra of a) Si@p(NIPA-co-APMA) and b) Si@pNIPA-b-pAPMA nanocomposites. C: carbon; N: nitrogen; O: oxygen; Fe: iron; Br: bromide; Si: silica.

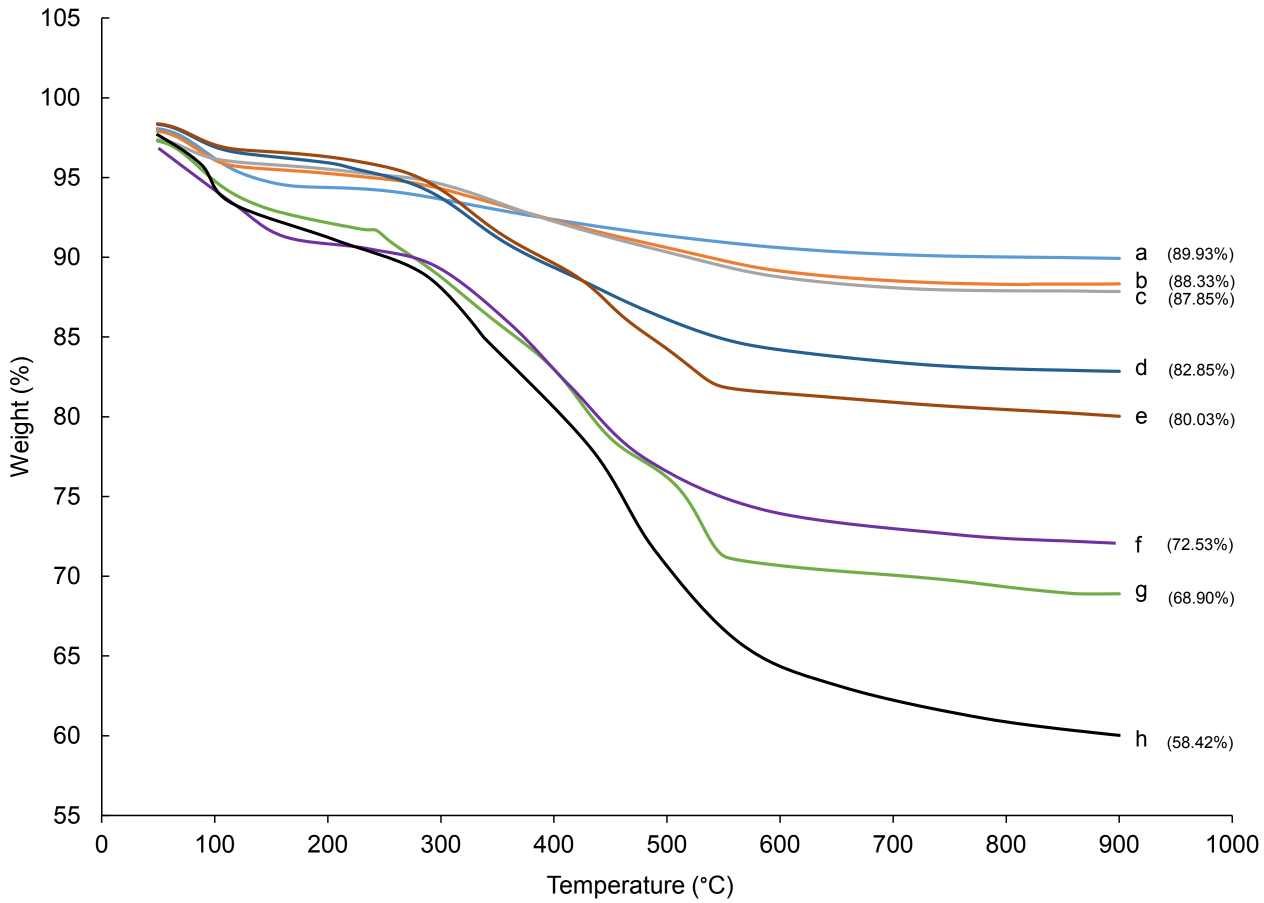

Figure S7. TGA analysis of the (a) silica core, (b) Si@NH_2, (c) Si@initiator, (d) Si@pNIPA, (e) Si@p(NIPAm-co-APMA), (f) Si@pNIPA-b-pAPMA, (g) Si@p(NIPA-co-APMA)$_{BA}$ and (h) Si@pNIPA-b-pAPMA$_{BA}$ particles.

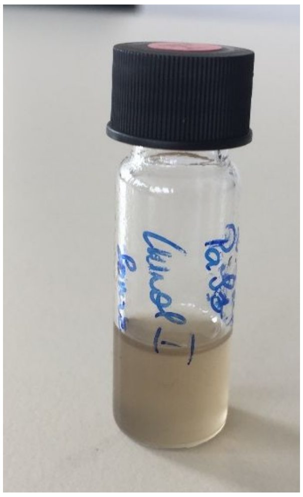

Figure S8. Resuspension of pNIPA cleaved from Si@pNIPA particles in DMF.

Photographer: Univ.-Prof. Dr. Oliver Brüeggemann, Head of the Institute of Polymer Chemistry, Johannes Kepler University Linz, Austria

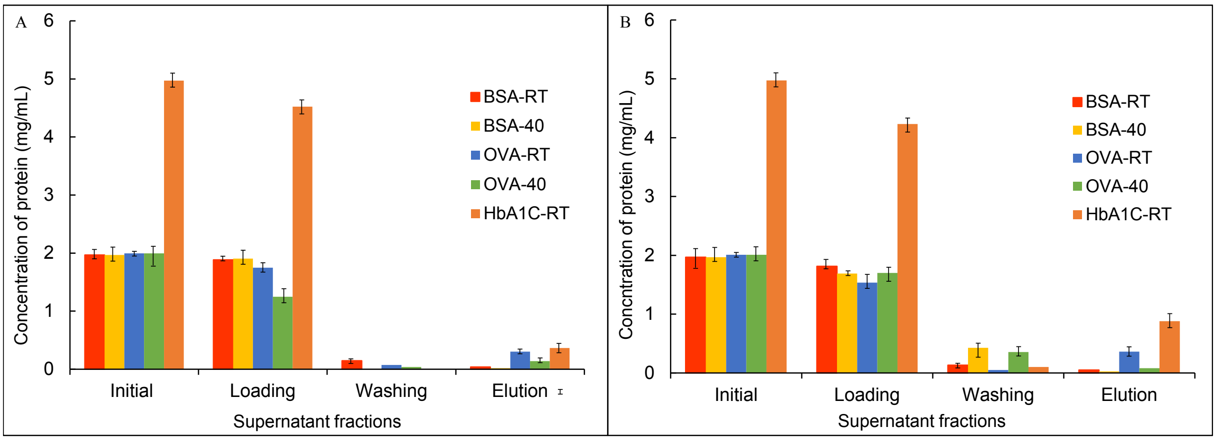

Figure S9. Concentrations of proteins from different steps of the adsorption/elution process at two temperatures (RT: 20 °C (room temperature) and 40: 40 °C); A) Si@p(NIPA-co-APMA)$_{BA}$ and B) Si@pNIPA-b-pAPMA$_{BA}$ nanocomposites. The initial concentrations of BSA, OVA and HbA1c were 2, 2 and 5 mg/mL, respectively.

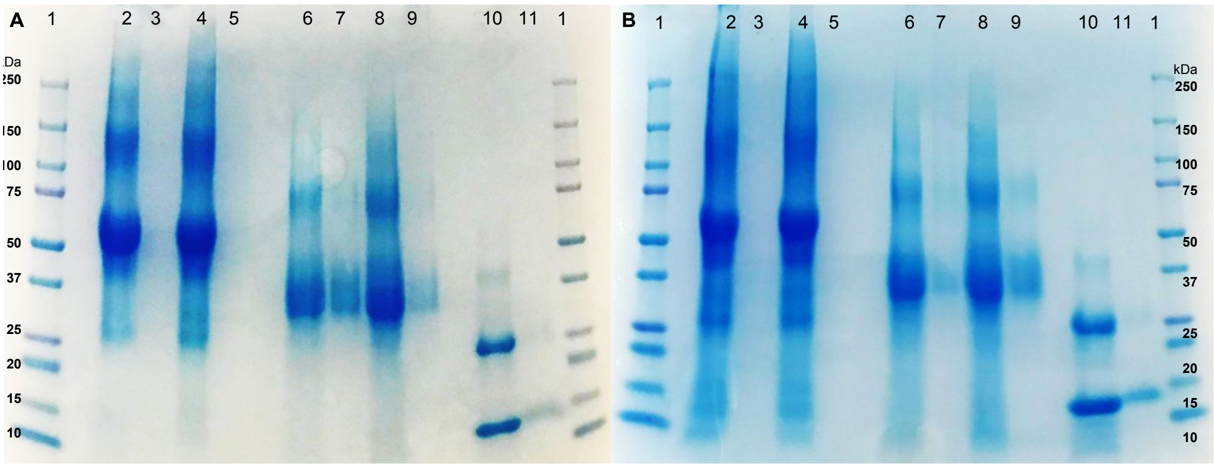

Figure S10. Digital images of SDS-PAGE analysis of protein samples tested on A) Si@p(NIPA-co-APMA)$_{BA}$ and B) Si@pNIPA-b-pAPMA$_{BA}$ nanocomposites. 1) Protein marker; 2) BSA solution loaded at 40 °C; 3) BSA eluted from nanocomposite related to #2; 4) BSA solution loaded at 20 °C; 5) BSA eluted from nanocomposite linked to #4; 6) OVA solution loaded at 40 °C; 7) OVA eluted from nanocomposite associated with #6; 8) OVA solution loaded at 20 °C; 9) OVA eluted from nanocomposite linked to #8; 10) HbA1c solution loaded at 20 °C; 11) HbA1c eluted from nanocomposite. All of the fractions were concentrated using a freeze-dryer prior to the analysis.

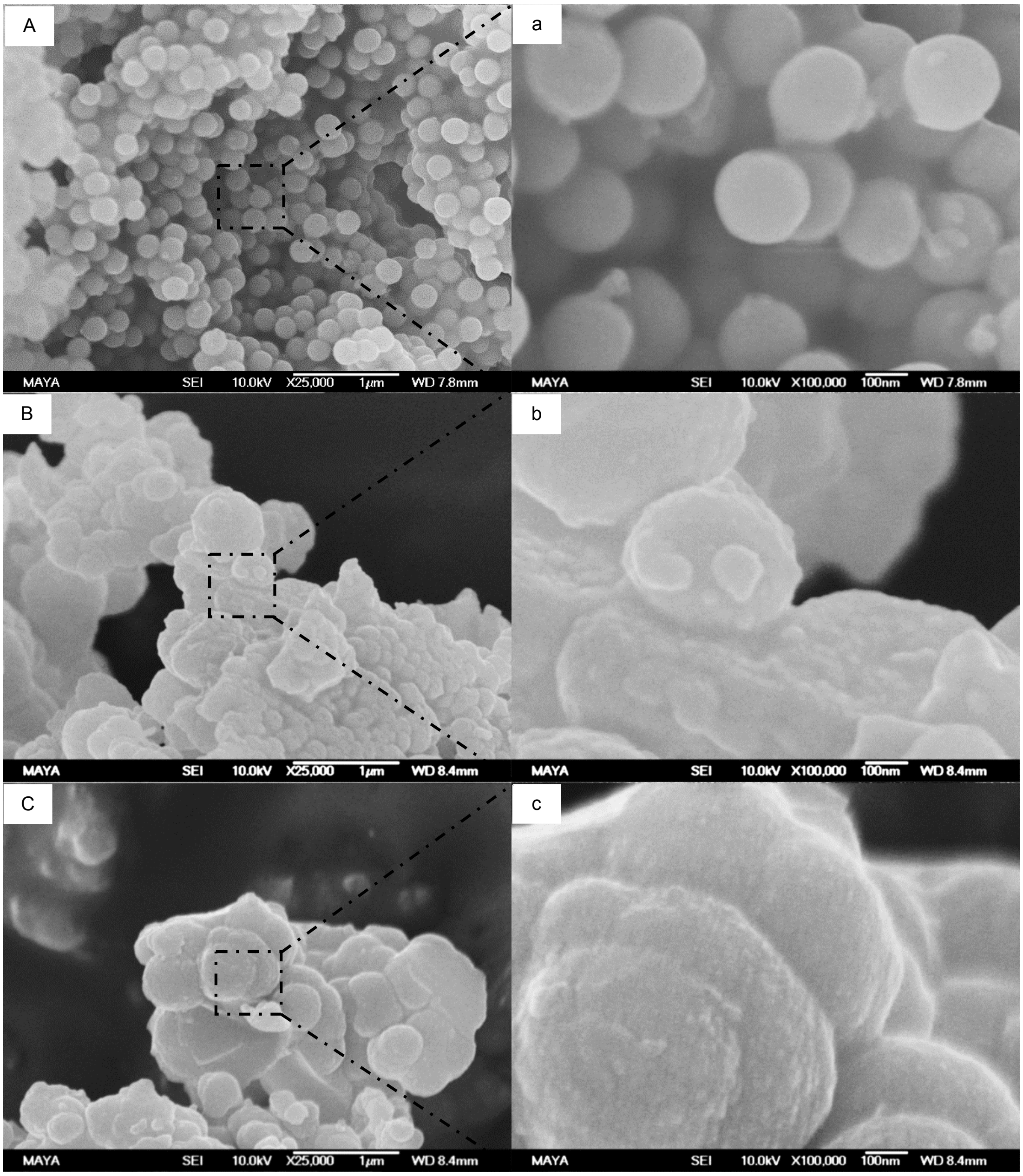

Figure S11. SEM images of A) Si@p(NIPA-co-APMA) nanocomposite; B) Si@p(NIPA-co-APMA)$_{ep}$ nanocomposite; C) Si@p(NIPA-co-APMA)$_{BA}$ nanocomposite (scale bar is 1 μm). Images a, b and c are the higher magnification of A, B and C, respectively, with a scale bar of 100 nm.

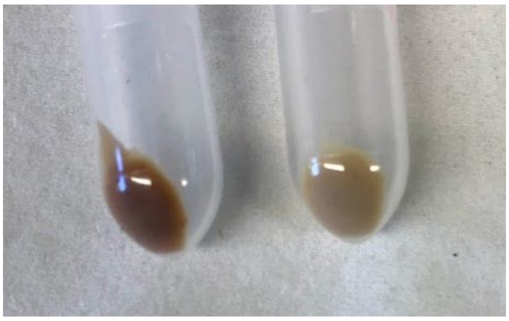

Figure S12. Digital photo of the nanocomposite (left) before and (right) after post modification with epichlorohydrin

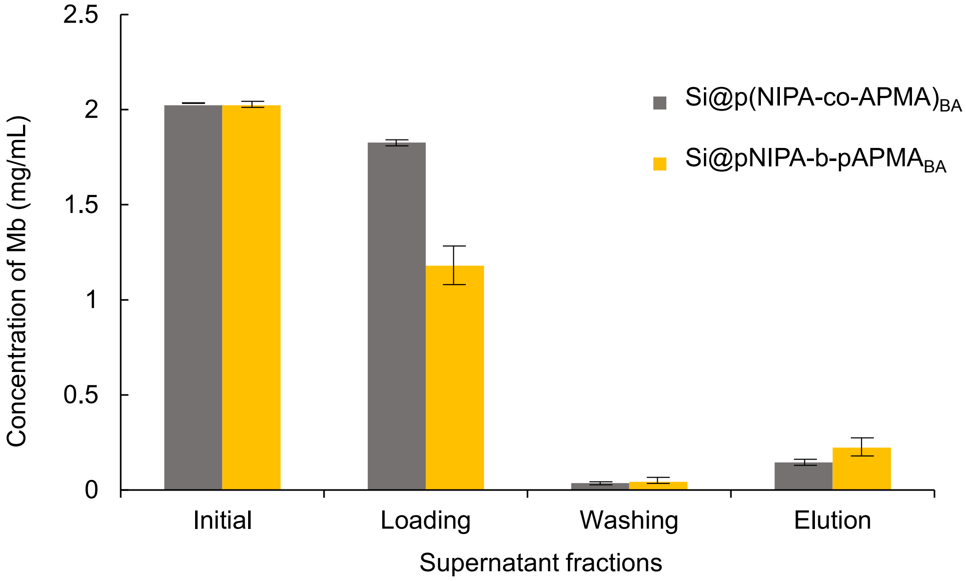

Figure S13. The concentration of Mb during the adsorption/elution process on Si@p(NIPA-co-APMA)$_{BA}$ and Si@pNIPA-b-pAPMA$_{BA}$ nanocomposites (at 20 °C and in phosphate buffer 0.1 M, pH 6). The initial concentrations of BSA and Mb were 4.1 and 2 mg/mL, respectively.

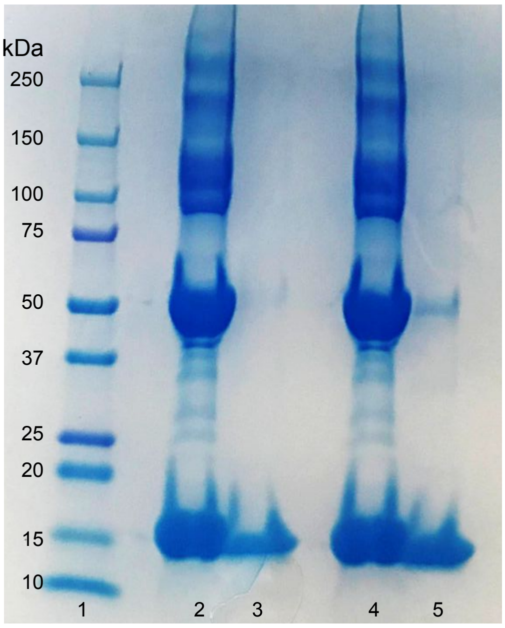

Figure S14. Digital image of SDS-PAGE analysis of protein samples tested on Si@p(NIPA-co-APMA)$_{BA}$ and Si@pNIPA-b-pAPMA$_{BA}$ nanocomposite.
1) Protein marker; 2) BSA and Mb solution loaded at 20 °C on Si@p(NIPA-co-APMA)$_{BA}$; 3) Elution fraction linked to #2; 4) BSA and Mb solution loaded at 20 °C on Si@pNIPA-b-pAPMA$_{BA}$; 5) Elution fraction linked to #4. All of the fractions were concentrated using a freeze-dryer.

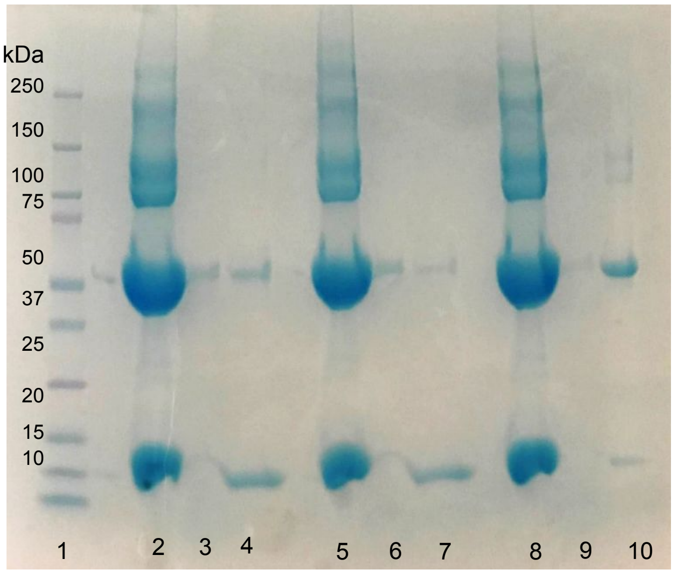

Figure S15. Digital image of SDS-PAGE analysis of protein samples tested on Si@pNIPA, Si@p(NIPA-co-APMA) and Si@pNIPA-b-pAPMA nanocomposites.
1) Protein marker; 2) BSA and Mb solution loaded on Si@pNIPA; 3) Washed fraction from Si@pNIPA; 4) Elution fraction from Si@pNIPA; 5) BSA and Mb solution loaded

on Si@p(NIPA-co-APMA); 6) Washed fraction from Si@p(NIPA-co-APMA); 7) Elution fraction from Si@p(NIPA-co-APMA); 8) BSA and Mb solution loaded on Si@pNIPA-b-pAPMA; 6) Washed fraction from Si@pNIPA-b-pAPMA; 7) Elution fraction from Si@pNIPA-b-pAPMA; All of the fractions were concentrated using a freeze-dryer.

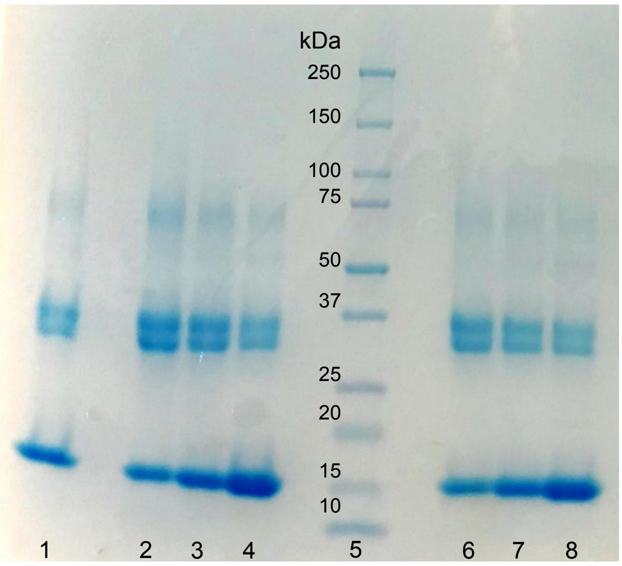

Figure S16. Digital image of SDS-PAGE analysis of protein samples tested on Si@p(NIPA-co-APMA)$_{BA}$ and Si@pNIPA-b-pAPMA$_{BA}$ nanocomposites. The initial concentration was 5 mg/mL for both Mb and OVA.

1) The original mixture before loading. 2) Supernatant separated from Si@p(NIPA-co-APMA)$_{BA}$ in pH 6 buffer. 3) Supernatant separated from Si@p(NIPA-co-APMA)$_{BA}$ in pH 7.5 buffer. 4) Supernatant separated from Si@p(NIPA-co-APMA)$_{BA}$ in pH 9 buffer. 5) Protein marker. 6) Supernatant separated from Si@pNIPA-b-pAPMA$_{BA}$ in pH 6 buffer. 7) Supernatant separated from Si@pNIPA-b-pAPMA$_{BA}$ in pH 7.5 buffer. 8) Supernatant separated from Si@pNIPA-b-pAPMA$_{BA}$ in pH 9 buffer.

Corresponding Author*

Solmaz Hajizadeh, Division of Pure and Applied Biochemistry, Department of Chemistry, Lund University, 22100, Lund, Sweden. E-mail: Solmaz.hajizadeh@tbiokem.lth.se, Telephone: +46 (0) 46 222 14 88, https://orcid.org/0000-0002-0348-8756

Authors

Leif Bülow, Division of Pure and Applied Biochemistry, Department of Chemistry, Lund University, 22100, Lund, Sweden https://orcid.org/0000-0003-4966-8610

Lei Ye, Division of Pure and Applied Biochemistry, Department of Chemistry, Lund University, 22100, Lund, Sweden. https://orcid.org/0000-0002-3646-4072
